# Neurodegeneration as Ecosystem Failure: A New Paradigm for Prevention and Treatment

**DOI:** 10.3390/ijms262211207

**Published:** 2025-11-20

**Authors:** Gordana Velikic, Gordana Supic, Dusica L. Maric, Miljan Puletic, Maja Ovcak Kos, Danilo Vojvodic, Dusan M. Maric

**Affiliations:** 1 Hajim School of Engineering, University of Rochester, Rochester, NY 14627, USA; ducamaric@gmail.com; 2 Department for Research and Development, Clinic Orto MD-P.A.R.K.S. Hospital, 21000 Novi Sad, Serbia; 3 Institute for Medical Research, Military Medical Academy, 11000 Belgrade, Serbia; gogasupic@gmail.com (G.S.); vojvodic.danilo@gmail.com (D.V.); 4 Medical Faculty of Military Medical Academy, University of Defense, 11000 Belgrade, Serbia; 5 Department of Anatomy, Faculty of Medicine, University of Novi Sad, 21000 Novi Sad, Serbia; 6 Faculty of Stomatology, Pancevo, University Business Academy, 26000 Pancevo, Serbia; miljenko.puletic@gmail.com; 7 Faculty of Law, University of Nova Gorica, 5000 Nova Gorica, Slovenia

**Keywords:** neurodegeneration, α-synuclein, exosomes, organ–brain axes, multi-pathogen synergy, environmental toxicants, synucleinopenia

## Abstract

Neurodegenerative diseases are widely viewed as brain-centric disorders defined by neuronal loss and protein aggregation. Yet decades of failed disease-modifying trials and mounting evidence of early peripheral symptoms suggest that this view is incomplete. This perspective review uses α-synucleinopathies as an illustrative example to outline how organ–brain axes, exosomal signaling, and strain competition shape disease trajectory, proposing a new clinical model: precision ecosystem medicine. This paradigm shift conceptualizes neurodegeneration as the late-stage manifestation of systemic ecosystem collapse: a progressive breakdown in inter-organ homeostasis driven by microbial imbalance, immune dysfunction, viral reactivation, co-infections, environmental stressors, and toxicant accumulation, when protective systems become saturated. Misfolded proteins such as α-synuclein act as prion-like mediators of this collapse, with distinct conformational “strains” emerging in different organ environments and may propagate to the brain via exosomes and neural pathways. Analyses suggest that understanding these systemic interactions could reveal new therapeutic windows before significant neurodegeneration occurs. This integrative approach establishes a conceptual foundation for regenerative therapies that address the complexity of neurodegenerative diseases beyond symptom management, offering promising directions for revolutionizing patient care with precise, multi-targeted strategies. Reframing neurodegeneration as a multi-organ, ecosystem-level disorder opens new paths for prevention, prediction, and potentially disease-modifying therapies, laying the theoretical foundation for a field of precision ecosystem medicine.

## 1. Introduction: From Brain-Centric Models to Ecosystem Failure

Neurodegenerative diseases represent one of the most significant biomedical challenges of our time. Multiple system atrophy (MSA), though rare, represents one of the most aggressive forms within the neurodegenerative spectrum, characterized by rapid progression, systemic deterioration, and fatal outcome within a decade of diagnosis. Despite decades of research investment and hundreds of clinical trials, no completely disease-modifying therapy exists for any of these disorders [1,2,3]. This persistent therapeutic impasse suggests not merely a methodological shortcoming, but a fundamental flaw in the prevailing conceptual models [4].

Traditionally, neurodegeneration has been framed as a brain-centric phenomenon, with pathology defined by hallmark protein aggregates: amyloid-β and tau in Alzheimer’s disease (AD), α-synuclein (α-syn) in Parkinsonian spectrum disorders (PSDs), TDP-43 in amyotrophic lateral sclerosis (ALS) and frontotemporal dementia (FTD) [5]. Therapeutic strategies have overwhelmingly targeted these aggregates or their formation within the central nervous system (CNS) [6]. Yet, the uniform failure of such approaches to alter disease trajectories highlights a missing dimension: these aggregates may not be isolated events in the brain, but rather the final manifestation of a longer, systemic process.

Mounting evidence now shows that neurodegeneration begins outside the CNS, often years before motor or cognitive symptoms emerge [7,8,9]. Constipation, orthostatic hypotension, REM sleep behavior disorder, reduced renal clearance, and periodontal disease can precede Parkinson’s disease (PD) motor onset by decades. Similarly, gut dysbiosis, viral reactivation, systemic inflammation, and metabolic stress have been implicated as early triggers in AD and ALS [10]. These observations suggest the existence of interconnected organ–brain axes whose continuous, bidirectional communication sustains protein homeostasis, immune balance, barrier integrity, and mitochondrial health. When these axes fail, pathology is no longer contained locally but propagates bidirectionally via neural tracts (e.g., the vagus nerve), endocrine mediators, immune signals, and extracellular vesicles (EVs) [11].

Within this framework, misfolded proteins such as α-syn may initially serve an adaptive role, sequestering toxic intermediates. But chronic systemic stress, microbial-immune disruption, and toxic exposures could progressively deplete the pool of functional proteins, tipping the balance into irreversible functional loss. In PD and related disorders, this process potentially culminates in synucleinopenia—a proposed state of depletion of functional α-syn but below the threshold required for vesicle cycling, mitochondrial stability, and barrier maintenance [12]. This transition from compensation to collapse represents the decisive turning point in the progression of disease.

Thus, neurodegeneration is best understood as ecosystem failure: the collapse of interconnected physiological networks that typically protect against protein misfolding and sustain resilience across the body. PSDs serve as the clearest case study of this phenomenon, with strong evidence linking peripheral dysfunction, misfolded protein propagation, and systemic failure [10]. Yet the same logic may be applied to other proteinopathies, where peripheral stressors, microbial ecosystems, and vesicle-mediated communication shape disease initiation and progression [11].

This review synthesizes converging evidence across neurodegenerative diseases to outline a unifying model of ecosystem collapse, structured around four interlocking themes: (i) the prion-like nature of misfolded proteins and their functional depletion, (ii) EVs and other transmission mechanisms, (iii) the “perfect storm” of microbial, viral, toxic, and inflammatory triggers, and (iv) the multi-axis organ–brain pathways that sustain or undermine resilience. Reframing neurodegeneration as the neurological endpoint of systemic failure highlights new opportunities for early detection, risk stratification, and precision ecosystem medicine aimed at preserving resilience before collapse becomes almost irreversible.

To guide the reader, the key terms used throughout this review are summarized in Box 1. Some of these are introduced here as entirely new conceptual framings (e.g., epigamilial susceptibility, epiglial scar), while others have appeared in prior literature in a fragmented or implicit way (e.g., vexosomes) but have lacked a standardized definition or systematic integration. This review aims to consolidate and formalize these terms within a coherent ecosystem framework, thereby enabling consistent usage across research and clinical contexts while acknowledging that these concepts await empirical validation.

Box 1 **Glossary of Terms Defining the Ecosystem Failure Paradigm.**

**Term**

**Definition**

**Relevance**
Perfect stormConvergence of microbial, viral, metabolic, circadian, immune, and toxic stressors that overwhelm resilience, leading to systemic collapse.Explains why neurodegeneration requires a multifactorial convergence of factors rather than a single cause, providing the theoretical foundation for multimodal therapeutic approaches and highlighting why isolated interventions typically fail.Molecular scarsUmbrella term for long-lasting imprints at epigenetic, proteostatic, mitochondrial, or microbial levels that sustain pathology even after triggers are removed.Captures why disease persists even when triggers are removed.Epiglial scarsMaladaptive reprogramming of glial cells (astrocytes, microglia, oligodendrocytes) that persists beyond the initial insult.Explain the persistence of neurodegeneration across an individual’s lifetime.Epigamilial SusceptibilityIntergenerational inheritance of pathogen- and environment-driven epigenetic marks reinforced by shared exposures.Explains familial clustering without clear genetic mutations.ProteinopeniaUmbrella term for functional depletion of essential proteins (α-synuclein, tau, TDP-43) below thresholds needed for cellular homeostasis, representing the actual tipping point across neurodegenerative diseases.Unifies the concept that disease emerges from loss-of-function rather than just toxic aggregation, generalizing across all major neurodegenerative disorders.SynucleinopeniaDepletion of functional α-synuclein below the threshold needed for vesicle cycling, mitochondrial stability, and barrier integrity.Defines the tipping point of Parkinsonian spectrum disorders.Tauopenia/TDP-43 depletionLoss of physiological functions of tau, or TDP-43 due to sequestration into aggregates, highlighting that disease is not only toxic gain-of-function but also loss-of-function.Generalizes the concept of functional collapse across diseases.Strain SelectionProcess by which ecological pressures favor specific conformational variants of misfolded proteins, determining disease phenotype and progression rate through "molecular Darwinism."Explains why similar protein aggregates produce different clinical syndromes (e.g., PD vs. MSA).Organ–brain AxesBidirectional communication pathways between peripheral organs and the brain that maintain homeostasis and can propagate pathology when disrupted.Central organizing concept for how systemic dysfunction spreads to neurodegeneration.VexosomesExtracellular vesicles carrying viral and other pathogen components, blurring the line between host communication and pathogen exploitation, representing hybrid host–pathogen messengers that propagate misfolding.Illustrate how pathogens hijack vesicle traffic to amplify misfolding.Trained ImmunityMaladaptive epigenetic reprogramming of immune and glial cells that maintains hyper-reactive inflammatory states long after initial pathogen clearance.Mechanistic basis for epiglial scarring and explains why neuroinflammation persists despite apparent pathogen elimination.Cascade DynamicsSelf-reinforcing feedback loops where interventions in one domain (epigenetic, microbiome, proteostatic, mitochondrial) propagate improvements across interconnected systems.Explains why strategic interventions may trigger disproportionate recovery effects through positive feedback mechanisms.Ecosystem Restoration/Precision Ecosystem MedicineTherapeutic approach targeting multiple organ–brain axes and pathogenic factors simultaneously to restore systemic resilience rather than treating isolated symptoms.Represents the clinical translation of the ecosystem failure paradigm into precision medicine strategies for the prevention and treatment of neurodegeneration.

## 2. Clinical Overlap as a Window into Systemic Convergence

The early stages of synucleinopathies such as PD and MSA often display overlapping motor, autonomic, and sleep-related symptoms, leading to frequent diagnostic uncertainty [13,14,15,16,17]. Rather than being viewed solely as a limitation of current clinical tools, this overlap reflects shared molecular and systemic processes that blur traditional disease boundaries. Post-mortem and biomarker studies reveal partial convergence in α-syn strain behavior, peripheral autonomic involvement, and inflammatory signatures, suggesting that these disorders may represent different manifestations within a continuous network of neuro-systemic dysfunction. A concise comparison of overlapping clinical features among major synucleinopathies is provided in Supplementary Table S1. 

Recognizing such clinical and biomarker intersections reframes diagnostic difficulty as evidence of underlying biological interdependence. The persistence of mixed phenotypes and evolving diagnostic criteria highlights that neurodegeneration does not unfold in isolation within the brain, but rather through distributed, organ-to-organ interactions. This systemic convergence, rather than symptom similarity per se, provides the rationale for approaching PD and related conditions through the lens of ecosystem failure. Understanding this continuum requires shifting from descriptive diagnostics toward mechanistic integration, explored in the following section on prion-like misfolding and functional depletion.

## 3. Prion-like Misfolding and Functional Depletion

A unifying feature across neurodegenerative diseases is the abnormal behavior of otherwise essential, proteins. α-Syn, tau, TDP-43, amyloid-β (Aβ), each exist in a homeostatic balance between functional and aggregation-prone conformations [1,18,19]. In contrast, monogenic disorders such as HD involve toxic gain-of-function mutations with secondary ecosystem effects. Under physiological conditions, these proteins support critical cellular processes: α-syn regulates synaptic vesicle cycling and mitochondrial integrity; tau stabilizes microtubules and neuronal transport; TDP-43 maintains RNA processing and nuclear homeostasis; and huntingtin participates in vesicle trafficking and transcriptional control. Disease arises when cellular stressors and aging shift this equilibrium toward pathological conformations, initiating a cascade of misfolding, aggregation, and cellular dysfunction. Cells counter with chaperones, lysosomal degradation, autophagy, and the ubiquitin-proteasome pathway, but these defenses, though initially effective, are gradually eroded and ultimately overwhelmed by cumulative stressors.

In addition to α-syn, tau, and TDP-43, several other aggregation-prone proteins exhibit comparable prion-like features. FUS and SOD1, for example, are central to certain forms of amyotrophic lateral sclerosis (ALS) and frontotemporal lobar degeneration (FTLD) [20,21,22,23,24]. C9orf72-derived dipeptide repeat proteins, polyglutamine-expanded ataxins, and the canonical prion protein in transmissible spongiform encephalopathies also display templated misfolding and strain diversity [20,25,26]. These examples demonstrate that prion-like propagation represents a generalizable biological response to proteostatic stress, extending beyond a few classical proteins and reinforcing the view that neurodegenerative diseases share a common molecular ecology of misfolding and functional loss [27,28].

These pathogenic proteins share prion-like properties: once a fraction becomes misfolded, the abnormal conformation can template native proteins to adopt the same state. Aggregates then propagate along neuronal pathways, across synapses, and even between organs, often via EVs or direct neural conduits. Importantly, not all aggregates are identical. Distinct strains or conformational variants of α-syn and tau have been identified, each with unique seeding potential, regional tropism, and clinical phenotype. The strain diversity helps explain why PD, MSA, PSP, or LBD, though all involving α-syn or tau, present with divergent patterns of neurodegeneration and symptomatology. The prion-like nature of α-syn aggregates has been demonstrated through transmission studies. Experimental studies have shown that the intracerebral injection of α-syn aggregates from MSA patient brains induces MSA-like pathology in transgenic mice, while transfected human embryonic kidney cells propagate similar pathology [19]. α-Syn from patient Lewy bodies also propagated pathological activity in cultured neurons [29], supporting the prion-like spreading hypothesis across different synucleinopathies. EVs isolated from cerebrospinal fluid (CSF) of PD patients induced α-syn aggregation in vitro, with misfolded and oligomeric α-syn species being preferentially sorted into EVs compared to native forms. Plasma-derived EVs from PD patients trigger α-syn aggregation in vivo when injected into wild-type mice [30], while microglia/macrophage-derived CD11b-positive EVs from PD patient CSF induce α-syn aggregation in vitro [12]. These findings reinforce the dual role of EVs as clearance mechanisms and vehicles for pathological propagation across the organ–brain axes. These findings support the hypothesis that α-syn assumes a prion-like behavior, suggesting MSA, PD, or LBD can all be considered prion-like disorders.

**Figure 1:**
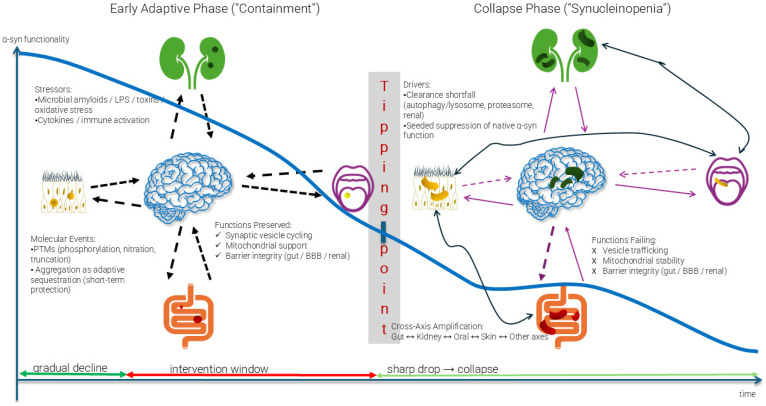
The Path to Synucleinopenia and Ecosystem Collapse. The early adaptive phase shows preserved α-syn functionality despite increasing stressors (microbial amyloids, toxins, oxidative stress). Molecular events, including post-translational modifications and adaptive aggregation, initially provide short-term protection. At the tipping point, clearance mechanisms fail, leading to the collapse phase characterized by synucleinopenia—functional α-syn depletion below critical thresholds. Cross-axis amplification then propagates dysfunction from initial sites (illustrative examples shown: gut, kidney, oral, skin) to other organ–brain axes, culminating in irreversible systemic failure. The intervention window represents the critical period preceding the tipping point, during which ecosystem restoration remains feasible. Arrow legend: Blue curve: α-syn functionality over time (gradual decline → tipping point → sharp collapse); Dashed black arrows: Stressor inputs into the CNS during the adaptive phase; Solid black arrows: Cross-axis amplification pathways transmitting dysfunction between organ-organ systems; Dashed purple arrows: Bidirectional prion-like propagation of misfolded species between organs and brain; Solid purple arrows: Reinforcement loops that maintain collapse once synucleinopenia is established; Dark green arrow (baseline): Gradual decline trajectory; Red arrow (baseline): Intervention window, where therapeutic reversal is possible. Light Green arrow (baseline): collapse—synucleinopenia phase.

Beyond aggregation, an equally important dimension is functional depletion. In the PSDs, early aggregation may potentially serve an adaptive role, sequestering toxic oligomers. However, chronic systemic stress may progressively exhaust the pool of functional α-syn, potentially leading to a state of synucleinopenia- a proposed concept describing the depletion of native α-syn below the threshold required to sustain vesicle cycling, mitochondrial stability, and barrier maintenance (Table 1). This definition builds on the neuronal loss-of-function model [12], extending it from cellular α-syn depletion to a proposed systemic framework in which proteinopenia acts as a tipping point for ecosystem collapse. The path to synucleinopenia can be understood as proceeding through an adaptive containment phase followed by a collapse phase (Figure 1). The accumulation of misfolded proteins results from upstream disruption of protein degradation pathways and protein quality control systems [31,32,33]. Chaperones maintain protein homeostasis through folding nascent polypeptides, refolding misfolded proteins, and directing terminally misfolded proteins toward degradation via the ubiquitin-proteasome system (UPS) or chaperone-mediated autophagy (CMA) [33]. While some aggregate components, such as ubiquitin, represent tagging for degradation rather than intrinsic toxicity, persistent proteostatic overload prevents their clearance, promoting the accumulation of misfolded species. Inflammatory cytokines such as TNF-α and IL-1β suppress autophagy flux. Oxidative stress depletes chaperone cofactors and reduces ATP availability for protein quality control, creating conditions where even robust cellular defenses become quantitatively insufficient.

When misfolded proteins escape targeted degradation, they form aggregates through hydrophobic interactions, reaching a tipping point that marks the transition from adaptive compensation to irreversible collapse, as illustrated in Figure 1. In its early phase, α-syn aggregation may act protectively—sequestering toxic intermediates and preserving essential cellular functions [34,35,36,37]. However, sustained stressors, including microbial amyloids, oxidative damage, and post-translational modifications, gradually exhaust the functional α-syn pool. Once this depletion crosses a critical threshold, the system enters the collapse phase characterized by synucleinopenia, where insufficient functional protein remains to maintain vesicle cycling, mitochondrial stability, and barrier integrity.

Beyond the cellular scale, this collapse propagates through a broader network, shifting from local containment to self-reinforcing decline, where cross-axis amplification spreads dysfunction from initial foci to interconnected organ systems. Misfolded species further accumulate, suppressing residual native protein function and magnifying vulnerability across organ–brain axes. Proteins involved in targeted degradation, such as heat shock protein 70 (Hsp70), the autophagy adaptor p62, and Sirtuins (SIRTs) [31,32], emerge as potential therapeutic targets for restoring proteostasis across the failing ecosystem.

The tipping point concept is supported by biophysical studies, suggesting that protein aggregation follows nucleation-dependent kinetics, where misfolded seeds may accelerate further misfolding in an autocatalytic manner. The transition from adaptive containment to collapse may represent the point at which seeding rates exceed clearance capacity, leading to self-sustaining pathological cascades [38]. This threshold behavior could explain why interventions may be highly effective before the tipping point, but progressively less effective afterward.

**Figure 2:**
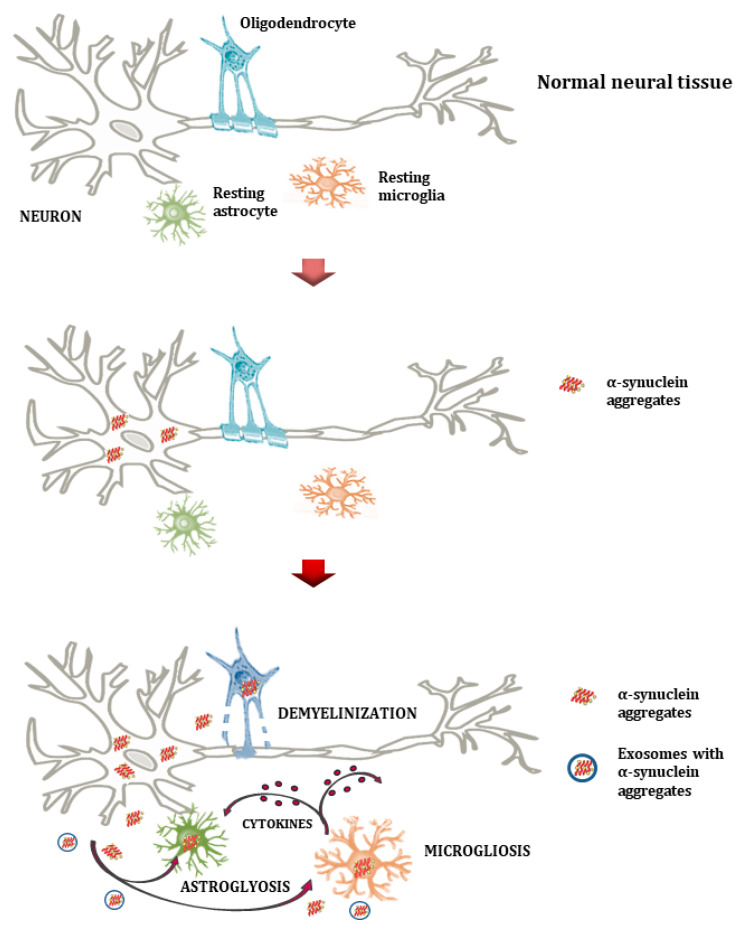
Normal neural tissue (**upper plot**) illustrates healthy interactions among neurons, oligodendrocytes, astrocytes, and microglia. Aggregates of α-syn form through the induced expression of the α-syn gene (SCNA) by various cells and/or the ineffective glial clearance mechanism (**middle plot**). Microglia and astrocytes phagocytize and accumulate misfolded α-syn. This results in oligodendrocyte degeneration, the loss of trophic support to neurons, and demyelination (**lower plot**). Extracellular α-syn aggregates cause astrocytosis and microgliosis, as well as the release of pro-inflammatory cytokines and reactive oxygen species, creating a neuroinflammatory milieu that further impairs neuronal integrity in PSDs. Microglia appear to have a dual role that involves the removal of α-syn and the production of cytokines.

**Figure 3:**
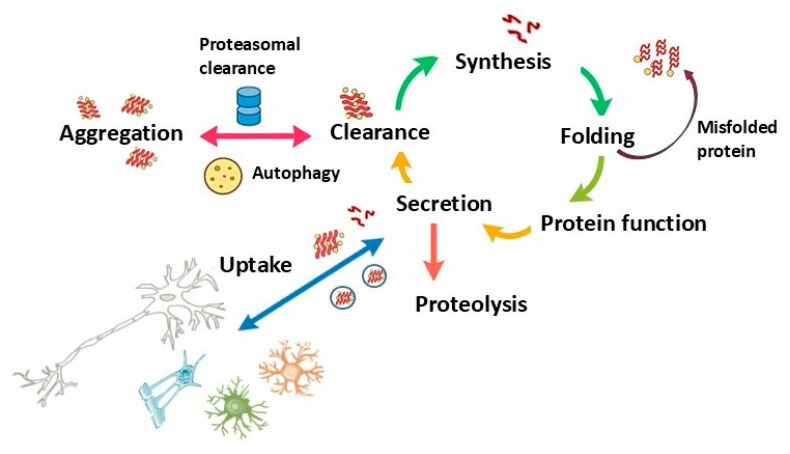
Protein Homeostasis Cycle: This schematic represents the cyclical processes involved in protein synthesis, folding, function, secretion, and clearance. Dysregulation at any point, particularly in folding, aggregation, or clearance, can lead to protein misfolding and aggregation, contributing to neurodegenerative processes. Uptake by surrounding cells highlights the potential for intercellular spread of misfolded proteins, a feature relevant in diseases such as MSA and PD, where protein aggregation and propagation exacerbate cellular dysfunction.

The mechanisms of α-syn accumulation in oligodendrocytes in MSA remain incompletely understood. Aggregates could form through the induced expression of the α-syn gene (SNCA) in oligodendrocytes and other glial cells under disease conditions, or result from oligodendrocyte uptake of α-syn secreted by neurons (Figure 2) [39]. The cellular milieu appears critical in different synucleinopathies. In MSA, oligodendrocytes might be more prone to accumulating neuron-derived α-syn than neuronal cells, possibly due to ineffective clearance mechanisms. More broadly, the accumulation of misfolded proteins results from upstream disruption of protein degradation pathways and protein quality control systems. Under normal conditions, cells maintain α-syn homeostasis through multiple interconnected degradation pathways (Figure 3). However, these clearance mechanisms in PSDs may become overwhelmed or dysfunctional, leading to pathological accumulation. The exosomal pathway is particularly significant within the ecosystem failure model, as it serves dual roles: functioning as a clearance mechanism while potentially contributing to the spread of pathological proteins between cells and across organ–brain axes.

Analogous processes are observed in other neurodegenerative diseases. In AD, hyperphosphorylated tau forms neurofibrillary tangles, but equally significant is the loss of soluble, functional tau, impairing axonal transport and synaptic stability [40,41]. In ALS and FTD, the cytoplasmic aggregation of TDP-43 coincides with nuclear clearance, thereby depriving cells of critical RNA-regulatory functions [42,43]. In HD, the widespread burden of mutant huntingtin creates systemic toxicity, with aggregates identified not only in the brain but also in skeletal muscle, pancreas, and heart [44,45,46]. Although monogenic neurodegenerative disorders, such as HD, arise from single-gene mutations, they converge onto the same systemic communication routes—EV trafficking, neuroimmune crosstalk, and peripheral organ involvement—highlighting that ecosystem failure represents a shared endpoint rather than a unique etiology. Across these disorders, pathology reflects a dual mechanism: toxic gain-of-function from aggregates (misfolded oligomers, fibrils, inclusions) and loss-of-function due to depletion of native proteins (synucleinopenia, tauopenia, etc.) [40,47,48]. While genetic mutations provide deterministic entry points into neurodegeneration, idiopathic counterparts arise from stochastic environmental insults. Yet, both converge upon the same inter-organ axes of communication. The eventual collapse, what is termed the “perfect storm,” emerges when genetic vulnerability, inflammatory signaling, and microbial or metabolic perturbations align across these axes, transforming adaptive communication into self-amplifying degeneration. 

**Table 1:** Overview of α-Syn’s Role in α-Synucleinopathies [49,50]. This table summarizes the production, propagation, and impact of α-syn within the brain and peripheral tissues, contrasting regular and misfolded forms, detailing the mechanisms by which α-syn aggregates cause cellular damage, and outlining therapeutic targets and diagnostic relevance. The comprehensive approach aims to provide insights into the multifaceted influence of α-syn on neurodegenerative pathophysiology and the potential avenues for clinical interventions and assessments.

Category	Details
α-Syn Production and Role	Produced primarily by neuronal cells, and to a lesser extent, by oligodendrocytes, neuroendocrine, and enteroendocrine cells. Its physiological role involves the regulation of neurotransmitter release at synapses and vesicle trafficking.
Role of Exosomes in Spreading	Exosomes, small vesicles released by cells, can carry α-syn, including its misfolded forms, between cells. This is believed to contribute to the propagation of pathology across different parts of the brain and potentially the peripheral nervous system.
Misfolded vs. Regular α-Syn	Regular α-syn is soluble and typically found in a non-aggregated state. Misfolded α-syn forms insoluble aggregates known as Lewy bodies (in neurons) or GCIs (in oligodendrocytes). Misfolded α-syn is prone to forming beta-sheet-rich structures that promote further aggregation.
Impact of α-Syn Aggregates on the Neural System	α-syn aggregates lead to cellular dysfunction and death via mechanisms such as mitochondrial disruption, impaired protein degradation pathways, and activation of inflammatory pathways, ultimately resulting in neurodegeneration.
Mechanisms of Cellular Damage	α-syn aggregates impair mitochondrial function, leading to reduced ATP production and increased oxidative stress. They also disrupt the ubiquitin-proteasome and autophagy-lysosomal pathways, crucial for protein degradation, and provoke endoplasmic reticulum stress, contributing to cell death.
Cellular Defense Failure	Threshold-dependent overload of protein quality-control systems. Inflammation impairs autophagy and proteasome function, while energy depletion reduces chaperone capacity. Synergistic co-stressors create vulnerability windows.
Therapeutic Targets	Therapies focus on reducing α-syn production (e.g., gene silencing), enhancing misfolded α-syn clearance (e.g., immunotherapy), potentially followed by healthy or therapeutic EV delivery, inhibiting its aggregation (e.g., small molecule inhibitors), and neuroprotective strategies to shield neurons from α-syn toxicity (e.g., antioxidants). However, challenges such as the blood–brain barrier, which limits the efficacy of drug delivery, remain significant obstacles to the development of effective treatments.
Diagnostic Relevance	Elevated levels of α-syn in CSF and blood, or its radiological markers, are being researched as potential diagnostic and prognostic biomarkers for synucleinopathies. Efforts to correlate these levels with disease severity and progression are ongoing. Variability in α-syn levels due to technical and biological factors poses challenges for biomarker development, making it difficult to correlate these levels consistently with disease severity and progression. Future work should systematically characterize defense thresholds and co-stressor effects on protein clearance.

This duality reframes neurodegeneration as a systemic crisis of proteostasis. Misfolded proteins act as seeds, amplifying pathology in a prion-like manner, while progressive loss of functional protein erodes cellular resilience. Together, these forces dismantle interconnected networks of neuronal, glial, and peripheral cell homeostasis, setting the stage for multi-axis propagation and ultimately leading to ecosystem collapse. 

## 4. Transmission Routes

The propagation of neurodegenerative pathology is not confined to individual neurons or even to the CNS. Instead, pathological proteins exploit multiple communication routes that link peripheral organs with the brain. These include EVs, neural tracts such as the vagus nerve, endocrine mediators, and immune pathways. Together, they create a multidirectional network in which local lesions can be amplified into systemic dysfunction.

### 4.1. Extracellular Vesicles as Double-Edged Messengers

Among these routes, EVs, including exosomes and microvesicles, are increasingly recognized as central mediators of disease spread. Under proteostatic stress, cells may release misfolded proteins packaged in vesicles as a form of compensatory clearance, sometimes referred to as “exophagy.” While this could lower intracellular toxicity, the vesicles appear to be enriched for oligomeric species with high seeding competence, effectively turning a local safeguard into a vehicle for systemic propagation [51]. This interpretation suggests that pathology arises not because protective functions are absent, but because they may be distorted under chronic stress, possibly shifting vesicle cargo away from clearance roles. This distortion does not occur uniformly; the transition from protective to pathogenic cargo depends on the cellular stress state and pathogen burden, thereby providing a mechanistic link between environmental challenges and systemic protein propagation. This dual role is supported by experimental evidence showing that EVs from PD patients induce α-syn aggregation when transferred to healthy cells and animal models, while simultaneously serving as natural clearance mechanisms under non-pathological conditions.

#### Cellular Defense Against Pathogenic Vesicle Cargo

Healthy recipient cells are not passive targets of EVs but employ multilayered protection systems that determine whether vesicular exchange remains homeostatic or becomes pathogenic [52]. Endocytic sorting channels potentially harmful cargo toward lysosomal degradation, while autophagic flux clears misfolded or aggregated proteins that escape vesicular compartments [53]. The ubiquitin-proteasome system further dismantles cytosolic aggregates and relieves proteostatic stress. At the plasma membrane, tetraspanin networks and pattern-recognition receptors (PRRs) regulate vesicle docking and uptake, acting as selective gatekeepers [54,55]. Under physiological conditions, these systems preserve cellular integrity. However, chronic inflammation, metabolic dysfunction, or pathogen exposure can saturate or reprogram these defenses, converting protective signaling into a pathogenic conduit for the transfer of misfolded proteins. Thus, vesicle-mediated propagation reflects not only the toxicity of transmitted cargo but also the failure of cellular clearance and recognition systems that ordinarily constrain its spread [56,57].

EVs also carry pathogen-derived cargo, including viral proteins, bacterial components, and toxins, which allows microbes to reprogram remote tissues and lower the threshold for protein misfolding. This hybrid role has led to the concept of vexosomes, here defined as vesicles incorporating viral material that blur the line between host communication and pathogen exploitation [58]. In this sense, EVs form a molecular postal system, binding disparate organ ecosystems into a shared vulnerability. Distinguishing protective from pathogenic vesicle traffic is not simply academic but represents a critical therapeutic target.

### 4.2. Neural, Endocrine, and Immune Pathways

Exosomes and EVs provide one mean of transmitting misfolded proteins and inflammatory signals across organ–brain boundaries. Beyond vesicle-mediated transport, protein aggregates spread through neural conduits, particularly the vagus nerve. This route has been implicated in the classic Braak hypothesis, and recently refined by Borghammer et al., where α-syn pathology ascends from the enteric nervous system and olfactory bulb to the midbrain [59,60]. Epidemiological studies support this model, showing that truncal vagotomy reduces PD’s risk decades later. Descending tracts also allow bidirectional flow, returning misfolded seeds from the brain to peripheral tissues and reinforcing systemic collapse.

Recent work further demonstrates that CSF flows beyond the CNS into peripheral nerves, reaching endoneurial and axoplasmic compartments [61]. This finding unifies central and peripheral nervous compartments into a single fluid-connected ecosystem, suggesting that misfolded proteins or inflammatory mediators may spread along CSF-peripheral nerve routes as well as through vesicles or vascular channels. Vascular channels also act as systemic conduits [62]. Circulating microbial products, cytokines, and EVs can cross the blood–brain barrier (BBB) through regions of fenestrated endothelium, active transport mechanisms, or barrier disruption during inflammation [57]. Small-vessel pathology further amplifies this route: endothelial dysfunction and basement membrane thickening, common in MSA and PD, facilitate leakage of inflammatory mediators and vesicles into neural tissue [63]. Thus, vascular routes provide a complementary mechanism of organ–brain communication that synergizes with neural and vesicular pathways.

Endocrine pathways provide a second axis of communication, where stress hormones, metabolic signals, and inflammatory cytokines prime distant organs for misfolding and immune dysregulation [64,65,66]. The immune system serves as a courier and amplifier: peripheral immune cells can engulf misfolded proteins or pathogen-derived EVs, cross the BBB, and release cargo into the CNS [67,68]. Chronic immune activation further destabilizes barrier integrity, compounding the spread of pathology.

### 4.3. Emerging Transmission Mechanisms

Recent advances have identified additional transmission routes that complement classical neural and vesicular pathways. Tunneling nanotubes enable direct cytoplasmic transfer of aggregates between distant cells [69], while the glymphatic system’s dual role in clearance and potential spread adds complexity to our understanding of CNS fluid dynamics [70]. Furthermore, strain-specific preferences for different transmission routes may explain why MSA-type aggregates favor systemic spread while PD-type aggregates remain more neurally confined [71].

Vascular channels represent another emerging route. Systemic cytokines, microbial fragments, and EVs can penetrate the CNS through a compromised or fenestrated BBB. Microvascular pathology, increasingly recognized as a hallmark of neurodegeneration, may transform the vasculature into a two-way conduit, allowing peripheral inflammatory signals and misfolded proteins to access neural circuits [72]. This vascular vulnerability may help explain why certain synucleinopathies, such as MSA, display broader systemic involvement: compromised vascular barriers facilitate aggregate entry and dissemination, whereas in PD, relatively intact vascular interfaces may restrict pathology to more neuronal routes.

### 4.4. Bidirectional Loops and Network Failure

Once misfolded proteins establish themselves within the CNS, they appear to rarely remain confined. Instead, they may participate in bidirectional loops, propagating pathology outward through descending fibers, vesicle traffic, or systemic immune signaling [73]. This explains why α-syn, tau, and TDP-43 aggregates are increasingly found in peripheral tissues such as, but not limited to, the gut, kidney, muscle, and skin. In these peripheral compartments, protective barriers and proteostatic defenses initially buffer against prion-like propagation. Still, once cumulative insults overwhelm these systems, misfolded proteins escape control and propagate through systemic loops. Each axis amplifies the vulnerability of others, creating a network-level collapse rather than a single-organ failure.

The question of whether neurodegeneration begins in the brain or the periphery has fueled ongoing debate [59,60]. Evidence supports both trajectories: in some individuals, early misfolded species appear first in peripheral tissues such as the gut, kidney, or oral cavity, while in others, especially genetic forms, pathology may initiate within the CNS. Unlike traditional body-first/brain-first models [60] that emphasize unidirectional spread from a single origin, the ecosystem failure framework recognizes that multiple axes may fail asynchronously, with rapid synchronization through bidirectional communication networks. Individual susceptibility factors, including genetic variants that affect barrier integrity, local pathogen burden (e.g., oral, gut, renal), and environmental exposures, likely determine which axis fails first and becomes the primary entry point for systemic propagation. Within the framework of ecosystem failure, however, the precise point of origin becomes less critical than the rapid establishment of bidirectional loops. Once seeded in either domain, misfolded proteins circulate through organ–brain axes via vesicular, neural, immune, and endocrine routes. This circulation erodes resilience synchronously across multiple systems, ensuring that local pathology is soon embedded within a network-level collapse. In this view, brain-first and organ-first models are not mutually exclusive, but may represent different entry points into the same systemic cycle of failure. Importantly, pathogens do not need to be physically present in the brain to influence disease; EVs can relay pathological information from peripheral reservoirs, amplifying neurodegeneration at a distance.

## 5. Pathogen Synergy and the Perfect Storm

Neurodegenerative diseases are increasingly recognized as the outcome of a synergistic interplay between pathogens, environmental exposures, and host vulnerabilities, rather than the linear consequence of a single agent [74]. This multifactorial convergence model may explain the long prodromal phase and the often abrupt transition to relentless decline once resilience thresholds are crossed. Within this framework, pathogens and their ecological interactions emerge as triggers of inflammation and as direct modulators of protein misfolding, prion-like propagation, and strain selection, amplifying systemic ecosystem failure. This convergence may be described as the “perfect storm”: a dynamic state in which microbial amyloids, viral reactivation, toxicant exposure, metabolic stressors, and immune dysregulation collectively overwhelm the body’s buffering capacity, tipping physiological systems from resilience to collapse.

### 5.1. Microbial Amyloids and Cross-Seeding of Host Proteins

Bacteria and fungi produce amyloid-like proteins that may act as conformational templates for host proteins. Curli fibers from *Escherichia coli* and amyloid-like fimbriae from oral pathogens accelerate the fibrillization of α-syn, tau, and amyloid-β by binding nascent intermediates and stabilizing β-sheet transitions [75,76]. These microbial amyloids are resistant to clearance and persist within biofilms, providing a constant source of amyloidogenic pressure. Animal studies confirm that colonization with curli-producing bacteria accelerates α-syn pathology, linking microbiome ecology directly to host misfolding. Thus, microbial amyloids do not merely provoke inflammation; they directly bias host protein folding kinetics, lowering the threshold for pathological aggregation.

### 5.2. Biofilms as Chronic Ignition Zones

At mucosal surfaces, such as the gut, oral cavity, and skin, microbes form multi-kingdom biofilms that commonly sustain barrier integrity and immune tolerance [77,78]. Under ecological stress, including antibiotic exposure, diet shifts, and pollutant exposure, beneficial taxa decline, and fungi such as *Candida albicans* undergo a commensal-to-pathogen transition, shifting from benign yeast to invasive hyphae that secrete proteases, β-glucans, and amyloid-like adhesins. Opportunistic bacteria, including *Streptococcus* and *Klebsiella*, exploit this weakened context, intensifying pro-inflammatory signaling [79,80]. Biofilms are adaptive ecosystems that evolve under immune and environmental pressure, becoming “living reservoirs” that continuously release amyloids, vesicles, and toxins into host tissues.

In this state, biofilms act as chronic ignition zones, maintaining low-grade, long-term exposure to amyloidogenic and inflammatory inputs, which prime host proteins, such as α-syn and tau, for misfolding.

### 5.3. Spatial Layering and Co-Infections in Bacterial and Viral Niches

The oral cavity exemplifies how pathogens occupy distinct tissue niches. Surface biofilms dominated by *P. gingivalis* and *T. denticola* release gingipains and amyloid-like proteins [81,82]. Beneath them, herpes viruses such as HSV-1, EBV, and CMV persist latently in different host reservoirs: HSV-1 in sensory neurons with epithelial reactivation sites, EBV primarily in B cells, and CMV in myeloid lineage cells. Periodic reactivation releases viral proteins and EVs, often by hijacking host exosome pathways (vexosomes), thereby amplifying systemic spread [83,84,85,86]. Deeper periodontal tissues harbor long-lived inflammatory scars, maintained by immune cells. This layered arrangement may create temporally staggered stress: bacterial products provide continuous exposure, viruses add intermittent “pulses” of pathogenic proteins, and immune scars could sustain chronic inflammation [87,88]. Such layering may ensure that the host proteome does not face isolated events, but rather overlapping waves of stressors, increasing the likelihood of protein misfolding and prion-like propagation [89]. When bacterial and viral co-infections coincide, these synergistic stress pulses can amplify systemic inflammation and vesicle-mediated spread, creating conditions particularly conducive to pathogenic protein misfolding and cross-seeding events implicated in PD, MSA, and related neurodegenerative disorders.

### 5.4. Commensal-Pathogen Transitions as Ecological Collapse

Many microbes exist on a continuum between beneficial and harmful roles, shifting their identity in response to the ecological context in which they reside. *Candida albicans*, a benign commensal under balance, becomes a pathogenic hyphal organism under metabolic or immune stress, releasing amyloid-like adhesins and toxins [90,91]. Similarly, *E. coli* can switch from a tolerated symbiont to a curli-producing, amyloidogenic strain, while even beneficial species such as *Akkermansia muciniphila* may, in excess, erode the mucus barrier, contributing to inflammation. These transitions do not represent isolated instances of microbial misbehavior, but rather a collapse of cooperative microbiome ecology into opportunistic behavior.

Candida overgrowth thus functions as a sentinel marker of broader ecological imbalance, often signaling parallel bacterial overgrowth (e.g., *Streptococci*) or depletion of protective taxa, such as lactobacilli. The consequence is increased exposure of host proteins to amyloidogenic and oxidative stress, conditions that favor pathological misfolding of α-synuclein, tau, and related proteins. Such co-dependency underscores why effective intervention requires comprehensive microbiome rebalancing rather than isolated antifungal therapy, which may transiently suppress fungi while leaving bacterial imbalance intact. In this sense, commensal-pathogen transitions exemplify a tipping point of ecological collapse, where systemic resilience is lost and neurodegenerative cascades are amplified.

#### Fungal and Parasitic Amplifiers in Multi-Pathogen Synergy

The pathogen consortium driving ecosystem failure extends beyond well-characterized bacterial and viral agents to include fungal and parasitic co-infections whose roles remain poorly defined but potentially significant. *Candida albicans* and other opportunistic fungi have been detected in postmortem brain tissues from patients with neurodegenerative diseases, although whether this represents causation, consequence, or methodological artifact remains disputed [92]. The neurotropic parasite *Toxoplasma gondii* offers a clearer mechanistic link, as chronic infection induces persistent microglial activation, alters dopamine metabolism, and creates tissue cysts that serve as reservoirs for sustained antigen exposure [93].

These agents may contribute to the perfect storm through several plausible mechanisms. Fungal cell wall components such as β-glucans and mannans activate complement cascades and pattern recognition receptors, potentially amplifying the inflammatory milieu that favors protein misfolding. *T. gondii* manipulates host cell metabolism and neurotransmitter systems in ways that could theoretically create conditions permissive for α-syn aggregation, though direct evidence for this pathway remains limited.

The relationship between bacterial dysbiosis and secondary fungal colonization illustrates the ecological succession characteristic of ecosystem collapse. Initial bacterial disruption-whether caused by antibiotic exposure, dietary shifts, or immune stress-creates inflammatory damage and altered metabolite profiles that favor fungal proliferation. This represents not an isolated pathogenesis but a cascading dysbiosis, where early bacterial imbalance creates niches that fungi exploit, thereby amplifying amyloidogenic pressure across organ–brain axes.

However, the clinical significance of these observations requires cautious interpretation. Antifungal trials in neurodegenerative patients have yielded mixed results, and the temporal relationship between infection and disease onset remains unclear in most cases. Within the ecosystem failure framework, fungal and parasitic agents likely function as amplifiers of an already disrupted ecology, rather than primary initiators, contributing to the multi-pathogen convergence that ultimately overwhelms host buffering capacity.

### 5.5. Viruses, Demyelination, and Oligodendroglial Vulnerability

Several neurotropic viruses exhibit tropism for oligodendrocytes and myelinated fibers, thereby directly compromising CNS structural integrity. *JC* virus in progressive multifocal leukoencephalopathy, *HHV-6* and *EBV* in multiple sclerosis, and reactivated herpes viruses in sensory ganglia all illustrate viral targeting of myelin. Loss of myelin impairs axonal conduction and destabilizes axonal metabolism, rendering neurons more susceptible to stress-induced aggregation [94,95,96,97]. Oligodendrocyte-derived exosomes, which are typically supportive of neuronal homeostasis, can be hijacked to deliver viral proteins or misfolded α-syn. This intersection of viral injury and vesicle hijacking biases ecosystems toward aggressive, glial-tropic protein strains, linking demyelination to the emergence of MSA- and PSP-like phenotypes.

### 5.6. Mast Cells and Microglia as Amplifiers of Co-Infection

Mast cells at barrier surfaces and meninges serve as hyper-responsive integrators of bacterial and viral signals [98,99,100]. Their degranulation releases histamine, proteases, and cytokines that degrade barriers and recruit microglia. Mast cells also shed inflammatory EVs, while microglia reciprocate with cytokine- and miRNA-loaded vesicles, establishing a mast cell-microglia feedforward loop. In mastocytosis-like states, even minor pathogen inputs could provoke disproportionate inflammatory cascades. This loop amplifies inflammation and generates oxidative stress (ROS, NO, peroxynitrite), which chemically modifies α-syn, tau, and TDP-43, making them more prone to aggregation [100]. These inflammatory mediators also compromise cellular quality-control systems: TNF-α and IL-1β suppress autophagosome-lysosome fusion, while reactive oxygen and nitrogen species impair proteasome activity and deplete chaperone cofactors [101]. As a result, recipient cells may lose the capacity to clear pathological proteins efficiently, not through passive capitulation but through progressive degradation of their defensive mechanisms.

### 5.7. Mitochondria as Signal Hubs, Not Just Victims

Mitochondria integrate multiple pathogenic signals: bacterial toxins, viral proteins, and cytokines. Pathogen interference with mitochondrial antiviral signaling (MAVS) undermines innate immunity, while oxidative phosphorylation failure reduces ATP for chaperone-mediated folding. Mitochondria release cardiolipin, mtDNA, and RNA as danger-associated molecular patterns (DAMPs), converting local stress into systemic immune activation. This dual role means that mitochondria are not passive casualties, but active hubs that translate microbial and inflammatory stress into systemic misfolding by exhausting proteostasis and chaperone capacity [102,103,104,105].

### 5.8. Strain Selection and Prion Biology

The diversity of disease phenotypes may reflect strain selection, a concept rooted in prion biology that could extend to other proteinopathies [106]. Classical studies of PrP have revealed that distinct conformations can be stably propagated, resulting in divergent clinical outcomes [107,108]. Analogously, α-syn, tau, and TDP-43 exist as conformational ensembles, and ecological pressures-such as microbial amyloids, inflammatory tone, and oxidative stress-select which strain proliferates. In PD, relatively benign strains may underlie slow progression, whereas in inflamed ecosystems, aggressive, MSA-like α-syn strains dominate, characterized by glial tropism and rapid seeding. Tau strains distinguish AD from PSP or CBD, while TDP-43 strains separate ALS from FTD.

This could be understood as “molecular Darwinism”: the strain best adapted to the host–pathogen ecosystem may survive and propagate, potentially linking pathogen ecology directly to the pace and phenotype of neurodegeneration. At the molecular level, different ecological conditions may favor distinct conformational intermediates: inflammatory cytokines could promote β-sheet-rich strains, oxidative stress may select for metal-binding variants, and specific microbial amyloids could template particular misfolding pathways. The strain that most efficiently exploits the local biochemical environment, whether neuronal in PD or oligodendroglial in MSA, becomes the dominant propagating species.

### 5.9. Molecular Scars and Trained Immunity

Pathogens may leave behind lasting imprints on host immune and glial cells, here conceptualized as molecular scars. Viral latency may produce recurrent PTMs on host proteins, while chronic bacterial exposure could alter DNA methylation and histone marks in immune and glial populations. These changes may induce “trained immunity”-a maladaptive epigenetic memory that could lock cells into hyper-reactive states [109]. Even after pathogen clearance, ecosystems remain biased toward inflammation and proteostatic failure. Such epiglial scars could ensure that once protein misfolding begins, the system cannot reset to a tolerant state, potentially amplifying chronic neurodegenerative trajectories. As elaborated in Section 7.5, Section 8, Section 8.1, Section 8.2, Section 8.3 and Section 8.4, ecosystem restoration strategies, including the coordinated modulation of inflammation, metabolism, and microbial balance, represent potential routes to reset these biased states and restore tolerance.

### 5.10. Environmental and Geographic Modulators

Environmental exposures further modulate this storm. Air pollutants, pesticides, and heavy metals, such as lead, mercury, cadmium, arsenic, and manganese, act as insidious co-triggers [110,111,112]. These polutants disrupt mitochondrial respiration, generate ROS, and directly bind cysteine/histidine residues on α-syn, tau, and TDP-43, destabilizing native folds. Heavy metals also reshape the microbiome, selecting for resistant and often amyloidogenic strains, thereby coupling environmental exposure to microbial ecology and misfolding pressure. Geographic variation in microbiomes and pollutant exposures may thus help explain regional heterogeneity in the incidence of neurodegenerative diseases.

### 5.11. Metabolic Cofactors and Circadian Disruption as Amplifiers of the Perfect Storm

Metabolic dysfunction and circadian misalignment add another critical layer to the synergistic pressures that drive neurodegeneration.

Diabetes, obesity, and metabolic syndrome generate chronic metabolic stress through hyperglycemia, insulin resistance, and lipid dysregulation [113,114]. These conditions accelerate the production of advanced glycation end-products (AGEs), increase mitochondrial ROS generation, and overload protein quality-control pathways, tipping proteostasis toward misfolding. Insulin resistance diminishes autophagy and proteasomal activity, thereby impairing the clearance of α-syn, tau, and TDP-43. At the systemic level, metabolic syndrome reprograms immune cells toward a pro-inflammatory phenotype, further destabilizing barrier integrity and fueling microglial hyper-reactivity. Epidemiological data consistently link type 2 diabetes and obesity to a higher risk of PD and AD, underscoring the metabolic contribution to systemic collapse.

Circadian disruption acts as a parallel temporal stressor [115,116]. Misalignment between central and peripheral clocks, resulting from shift work, jet lag, chronic sleep restriction, or light-at-night exposure, alters the timing of immune surveillance, hormone secretion, and microbiome activity [117]. Pathogens exploit circadian windows of vulnerability: herpes viruses and influenza viruses show circadian-dependent replication rates, while bacterial adherence and biofilm formation peak under disrupted rhythms. At the host level, clock gene dysregulation reduces mitochondrial resilience, impairs DNA repair, and exaggerates inflammatory responses. Sleep fragmentation also promotes glymphatic dysfunction, lowering the clearance of misfolded proteins from the brain interstitium.

Together, metabolic cofactors and circadian disruption serve as silent amplifiers of the perfect storm. They increase the probability that microbial amyloids, viral reactivation, toxin exposure, and inflammatory cascades converge, leading to irreversible proteostatic failure. By coupling energetic stress with temporal desynchronization, these factors create ecological niches where pathogens thrive, barriers leak, and host proteins misfold more readily, accelerating the collapse of resilience across organ–brain axes.

### 5.12. Synthesis: The “Perfect Storm” as Systemic Buffer Exhaustion

Taken together, microbial amyloids, biofilm transitions, bacterial-viral layering, commensal-to-pathogen shifts, viral myelin injury, mast cell-microglia loops, mitochondrial signaling, strain selection via prion-like Darwinism, molecular scars, and environmental stressors—a coherent picture emerges. For decades, the host proteome resists these pressures through proteostasis, immune tolerance, and barrier integrity. Eventually, cumulative hits may exhaust buffering capacity, leading to functional depletion states such as synucleinopenia, tau loss, and TDP-43 nuclear clearance. At this tipping point, protein misfolding may become irreversible and self-propagating, embedding pathology within organ–brain networks.

The “perfect storm,” therefore, reframes neurodegeneration as the endpoint of systemic ecological collapse, where microbes, viruses, toxins, pathogens, metals, and host responses converge to dismantle resilience and accelerate prion-like proteinopathies. Importantly, the mechanistic pathways connecting individual storm components to protein misfolding are increasingly supported by experimental evidence. Microbial amyloids have been demonstrated to cross-seed host proteins in vitro and in animal models. Viral proteins directly modify α-syn and tau through post-translational modifications. Heavy metals bind specific amino acid residues, destabilizing protein conformations. These individual mechanisms, while established, require integration studies to demonstrate their synergistic effects and cumulative thresholds in human disease progression.

The personalized nature of this convergence explains the clinical heterogeneity observed in neurodegenerative diseases. Rather than requiring identical mechanisms across all patients, the “perfect storm” model proposes that individual combinations of stressors determined by genetics, exposures, microbiome composition, and timing create unique vulnerability profiles that manifest as distinct disease trajectories. Despite this variability, these pathways converge on shared endpoints: protein functional insufficiency and ecosystem collapse.

The ecological convergence described in Section 5 does not remain diffuse but becomes anatomically inscribed along organ–brain axes. These axes act as gateways for external stressors and conduits for the propagation of misfolded proteins, transforming systemic vulnerability into spatially patterned neurodegeneration.

## 6. Organ–Brain Axes: Entry Points and Bidirectional Loops

The anatomical spread of neurodegeneration reflects the failure of multiple organ–brain axes rather than isolated CNS pathology [118]. These axes provide entry points for pathogens and toxins and may serve as potential outflow routes for misfolded proteins, possibly establishing bidirectional loops that entrench systemic disease. Crucially, failures are asynchronous: collapse in one axis (e.g., gut dysbiosis, kidney toxin overload) may precede others, creating heterogeneous clinical presentations. Yet, over time, these failures converge, producing a cumulative systemic collapse in which local pathologies become network-wide.

The systematic characterization of organ–brain axes in neurodegenerative diseases has advanced significantly through large-scale multicentric prospective studies. The Parkinson’s Progression Markers Initiative (PPMI), an international observational study conducted across nearly 24 sites [119]. This landmark study has documented peripheral biomarker changes that precede motor symptom onset by years, providing empirical validation for the multi-axis framework. Additional multicentric efforts examining gut–brain communication [10,120,121], cardiac denervation patterns, and peripheral α-syn pathology [122,123,124,125] have established that organ–brain axis dysfunction is not conjectural but represents a well-documented and systematically studied aspect of neurodegeneration. The following subsections detail the mechanistic basis and clinical implications of each major axis, building upon this empirical foundation. 

**The Gut–Brain Axis.** The gut is the most studied entry point, consistent with Braak’s hypothesis [59]. Dysbiosis alters the microbial composition, leading to an overrepresentation of amyloid-producing bacteria (e.g., curli-positive *E. coli*) and opportunistic fungi, such as *Candida albicans*. Emerging evidence suggests that viral infections, both acute and latent, may act as prime initiators of gut dysbiosis, disrupting epithelial integrity and mucosal immunity before bacterial and fungal shifts occur. These viral perturbations can induce α-syn expression in enteric neurons as part of innate antiviral defense, linking early virome activity to later protein misfolding cascades. Once dysbiosis is established, bacteria and fungi amplify pathology by generating amyloids, vesicles, and toxins that traverse compromised epithelial barriers [126]. Enteric neurons appear to accumulate α-syn aggregates decades before motor onset, which may ascend the vagus nerve to the brainstem. Epidemiological data showing reduced PD incidence after vagotomy strongly support this pathway.

Once CNS misfolding initiates, the loop may become bidirectional: descending tracts and CNS-derived exosomes could carry misfolded α-syn and inflammatory cargo back to the gut, potentially further destabilizing the microbiota and epithelial integrity. Biomarkers such as gut α-syn deposits, microbial EVs in stool, and vagal autonomic dysfunction are under active study.

The appendix has also emerged as a potential peripheral reservoir of α-syn. Aggregated and misfolded α-syn species have been detected in appendix tissue in patients with PD, and also in healthy individuals, underscoring that their presence alone is insufficient to initiate pathology [127]. Instead, the appendix may act as a long-term source of pathological seeds that, under conditions of impaired clearance or heightened inflammatory signaling, can enter gut–brain circuits and accelerate neurodegeneration [128]. Epidemiological studies reporting altered Parkinson’s risk following appendectomy further support the appendix as a modulatory site within the gut–brain axis [129]. Thus, the gut–brain axis may represent a microbial ignition site and a feedback loop, potentially reinforcing α-syn misfolding. These findings highlight how microbial dysbiosis and anatomical reservoirs contribute to long prodromal phases of disease, reinforcing the ecosystem failure paradigm. However, peripheral reservoirs, such as the appendix, can harbor misfolded α-syn even in healthy individuals; systemic stressors and clearance failure determine whether this becomes pathogenic. 

**The Oral–Brain Axis.** Periodontal disease provides a second ignition zone. *Porphyromonas gingivalis* and *Treponema denticola* release gingipains, amyloid-like proteins, and OMVs, which enter the bloodstream and cranial nerves [82]. Herpes viruses (*EBV*, *HSV*) persist latently in gingival tissues, creating a layered infection that amplifies the pressure of protein misfolding. Oral pathogens and viral reactivation appear to fuel systemic inflammation and may directly modify α-syn and tau via proteolysis and PTMs.

Cranial nerve connections (e.g., the trigeminal nerve) and vascular routes deliver these signals to the brain. Conversely, misfolded α-syn and tau may appear in salivary glands and oral mucosa, providing minimally invasive biomarkers [130,131]. The oral–brain axis highlights how layered microbial-viral niches and cranial connectivity integrate into systemic proteinopathy. 

**The Kidney–Brain Axis.** The kidney regulates systemic toxin clearance. Chronic kidney disease increases the circulation of uremic toxins, which destabilize proteostasis and promote oxidative stress. α-Syn misfolding has been observed in renal tissues, and EVs carrying misfolded α-syn and TDP-43 can travel from the kidney to the brain. Conversely, α-syn pathology has been described in renal nerves, suggesting bidirectional spread [132].

Heavy metals (cadmium, lead, mercury), which accumulate in the kidney, act as long-term reservoirs of toxicity, perpetuating systemic stress even after exposure ends. Kidney-derived EVs, circulating uremic toxins, and peripheral α-syn aggregates may serve as biomarkers. The kidney–brain axis underscores clearance failure as a trigger and an amplifier of misfolding stress [133]. 

**The Liver–Brain Axis.** The liver serves as a central hub for metabolism and detoxification. Dysregulation of lipid metabolism influences tau phosphorylation and α-syn aggregation, while impaired detoxification increases systemic oxidative burden [134,135]. Liver-derived EVs enriched in misfolded proteins and inflammatory mediators cross into the circulation, priming the CNS proteostasis collapse [136]. Viral hepatitis and systemic inflammation further strain this axis. Emerging evidence suggests that cholesterol metabolism, bile acid signaling, and microbiome-liver crosstalk are linked to neurodegenerative trajectories [137]. Blood-based liver metabolites and exosomes provide candidate biomarkers. The liver–brain axis highlights metabolic misregulation as a driver of systemic proteostatic instability. 

**The Heart–Brain Axis.** Autonomic dysfunction is a hallmark of PDs, particularly MSA. Cardiac sympathetic denervation often occurs early, sometimes before motor symptoms appear. Misfolded α-syn and tau are found in cardiac nerves and atrial tissue, reflecting systemic spread. Cardiac dysfunction compromises perfusion, impairing metabolic resilience in the brain [138,139,140]. Conversely, CNS pathology alters autonomic regulation, which in turn affects cardiac rhythm and vascular tone. Biomarkers include cardiac MIBG scans (showing denervation), ECG irregularities, and α-syn in atrial biopsies [141,142,143,144]. The heart–brain axis illustrates how autonomic failure and the spread of misfolded proteins are interwoven. 

**The Skin–Brain Axis.** The skin provides a unique diagnostic window. α-Syn and tau aggregates have been detected in cutaneous nerves, sweat glands, and fibroblasts, mirroring CNS pathology. Skin is also a site of microbial colonization and biofilm formation, with pathogens and EVs contributing to systemic inflammation [145]. Because skin is easily sampled, cutaneous α-syn and tau deposits are promising biomarkers. This axis demonstrates how misfolded proteins manifest at barrier sites, reflecting peripheral vulnerability and systemic spread.

**The Vagus–Brain Axis.** The vagus nerve is a conduit and a sentinel [146,147,148,149]. Aggregates of α-syn appear in vagal neurons and enteric plexuses early in disease, consistent with bottom-up propagation [147]. Truncal vagotomy reduces PD incidence, strongly implicating this pathway.

Importantly, vagus signaling is bidirectional: CNS misfolding propagates downward, altering enteric function, while peripheral misfolding propagates upward. This axis also integrates endocrine and immune signals, reinforcing its centrality [150]. The vagus–brain axis remains the archetype of prion-like neuronal propagation.

**Additional Organ–Brain Axes and Overlooked Pathways.** While this review focuses on the most substantiated organ–brain axes: gut, oral, kidney, liver, heart, skin, vagus, and immune, it is important to acknowledge that other systemic axes also contribute to shaping neurodegenerative risk. The lung–brain axis has been linked to hypoxia, systemic inflammation, and particulate matter exposure, which may accelerate protein misfolding. Endocrine–brain interactions, including thyroid and adrenal signaling, modulate metabolism, circadian rhythms, and immune tone [151]. The reproductive axis influences neuroprotection through sex hormones and may help explain gender differences in neurodegenerative prevalence and progression [152,153]. Musculoskeletal and bone–brain communication, mediated through myokines and osteokines, contributes to systemic resilience and mitochondrial health [154]. The bladder–brain axis encompasses the phases of urine storage and voiding, each of which requires precise coordination among the sympathetic, parasympathetic, and somatic nervous systems. The abnormal accumulation of α-syn can also occur in the peripheral autonomic nerves supplying the bladder, such as the pelvic and pudendal nerves [155,156]. This combination of central and peripheral neuronal pathology involving the brain–bladder axis is responsible for the various urinary symptoms observed in neurodegenerative disease patients. Recent studies suggest that the urinary tract may harbor a distinct microbiome, which could modulate local immune responses and impact autonomic bladder control. The larynx–brain axis is primarily mediated through neural, endocrine, and immune pathways, engaging brain regions and cranial nerves responsible for voice modulation and airway control. The vagus nerve, a key conduit in gut–brain communication, also innervates the larynx, raising the possibility that prion-like propagation of α-syn could extend along its laryngeal branches. Accumulation of α-syn within the nuclei of cranial nerves that control laryngeal function may directly impair motor control of voice and swallowing, while also affecting autonomic neurons, leading to dysphonia, voice changes, or even respiratory dysfunction in advanced cases [157]. The oropharyngeal and upper airway microbiome, including latent viral and bacterial communities, may further modulate vagal tone, aggravate mucosal inflammation, and compromise laryngeal resilience. Although the precise mechanisms of α-syn propagation along the brain–larynx axis remain unclear, emerging evidence supports its inclusion as an additional, underexplored pathway of systemic neurodegeneration. While these axes are less extensively characterized than the canonical gut–brain and vagus–brain pathways, increasing evidence supports their contribution to systemic neurodegeneration. Future research may reveal that they act as amplifiers or modifiers of the major axes described here, further reinforcing the systemic, interconnected nature of neurodegeneration.

### 6.1. The Immune–Organ–Brain Axis (Meta-Axis)

The immune system is not just a mediator but an independent communication channel between the periphery and the brain. Peripheral infections trigger cytokines (IL-1β, TNF-α, IL-6) that cross the BBB or signal via vagal afferents [158]. Monocytes and T cells infiltrate through the choroid plexus and meninges, carrying pathogen cargo or misfolded proteins.

Chronic infections reprogram immune cells via epigenetic scars (trained immunity), locking microglia into hyper-reactive states. Mast cells and macrophages release EVs enriched in inflammatory proteins and microRNAs, while CNS-derived misfolded α-syn and tau are exported in vesicles that circulate back to immune cells. This establishes immune-mediated bidirectional loops, making the immune–organ–brain axis a “meta-axis” that integrates signals from gut, oral, kidney, and liver ecosystems [5].

### 6.2. Converging Inflammatory Pathways: The Heart–Oral–Vascular–Brain Axis

While individual axes such as the heart–brain and oral–brain have been studied, mounting evidence suggests that many axes function as interconnected circuits rather than in isolation [159,160,161]. For illustrative purposes, we will explore the heart–oral–vascular–brain multi-axis connection.

The oral cavity and cardiovascular system share circulation, immune mediators, and pathways for microbial dissemination. Chronic periodontal disease not only drives systemic inflammation but also impacts vascular health and cardiac function, which in turn shape cerebral perfusion and neuronal resilience. Conversely, cardiac autonomic dysfunction and vascular compromise weaken immune regulation and barrier defenses, amplifying oral infections and systemic cytokine release [162]. This bidirectional interplay may create an integrated heart–oral–vascular–brain axis, in which insults arising in one domain could reverberate across the others, potentially reinforcing misfolding cascades and systemic collapse.

#### 6.2.1. Oral Infections as Upstream Triggers

Periodontitis, one of the most prevalent chronic infections, represents a persistent source of inflammatory stress. Pathogens such as *Porphyromonas gingivalis* and *Treponema denticola* release gingipains, amyloid-like proteins, and outer membrane vesicles (OMVs) that disseminate through the bloodstream and cranial nerves [87]. Herpes viruses, including HSV and EBV, establish latency within gingival tissues and immune cells, periodically reactivating to release bursts of proteins and vesicles [88]. These microbial and viral products enter circulation, elevate systemic cytokines such as IL-6, TNF-α, and IL-1β, and directly modify α-syn and tau through proteolysis and post-translational modifications (PTMs). The resulting systemic inflammatory tone lowers the threshold for protein misfolding and primes distant tissues for prion-like propagation.

#### 6.2.2. Vascular Pathology as a Converging Mechanism

The vasculature provides a direct highway linking oral inflammation with cardiac and cerebral outcomes. Periodontal disease has long been associated with endothelial dysfunction, atherosclerotic plaque formation, and increased cardiovascular risk. Microbial components such as lipopolysaccharide and gingipains can directly damage the vascular endothelium, while pro-inflammatory cytokines impair nitric oxide signaling, leading to reduced vasodilation and microvascular dysfunction. Notably, microvascular pathology in the heart (coronary microvascular angina) and brain (cerebrovascular small vessel disease) share key features: arterioles under 200 μm, precise autoregulation, basement membrane thickening, and vulnerability to oxidative stress [163,164]. This overlap suggests that systemic vascular compromise serves as a shared substrate connecting oral infections, cardiovascular dysfunction, and neurodegeneration. Recent evidence from AD’s research further supports this view: reduced levels of the vascular scaffolding protein CD2AP in brain endothelial cells are associated with impaired nutrient transport, cognitive decline, and increased pathology, particularly in men. These findings highlight vascular health as a critical determinant of neuronal resilience and suggest that microvascular pathology may act as a common gateway linking systemic stressors, metabolic disorders, and neurodegeneration [165].

#### 6.2.3. Cardiac Autonomic Dysfunction as Amplifier

In MSA and related synucleinopathies, cardiac sympathetic denervation often precedes the onset of motor symptoms [166,167]. Misfolded α-syn and tau are detectable in cardiac nerves and atrial tissue, reflecting systemic spread along autonomic circuits. Loss of autonomic regulation disrupts heart rate variability, baroreflex sensitivity, and vascular tone, compromising cerebral perfusion. Tissue hypoxia and impaired nutrient delivery exacerbate oxidative stress, mitochondrial failure, and protein misfolding in the brain. Conversely, systemic inflammation and dysbiosis increase cardiac vulnerability, reinforcing a vicious cycle of heart–brain–oral cross-talk. Biomarkers, such as reduced cardiac MIBG uptake, ECG irregularities, and α-syn deposits in atrial biopsies, highlight the mechanistic link between cardiac dysfunction and central proteinopathies.

#### 6.2.4. Ecosystem Failure Across Interconnected Systems

The interplay of oral infection, vascular inflammation, and cardiac autonomic dysfunction exemplifies the ecosystem failure paradigm:

Upstream ignition: oral pathogens and latent viruses produce continuous and pulsed inflammatory signals.

Systemic conduit: vascular endothelium transduces these signals into microvascular dysfunction in both heart and brain.

Amplification loop: cardiac denervation and perfusion deficits reinforce brain vulnerability, while systemic inflammation exacerbates oral disease and vascular stress.

Prion-like dynamics: microbial amyloids, viral vesicles, and modified α-syn/tau spread along cranial and vascular routes, seeding pathology in distant organs.

Together, this multi-axis network produces overlapping stress waves that converge on neuronal systems, reducing resilience and hastening collapse.

These observations support the existence of a distinct heart–oral–vascular–brain axis, where chronic infection, vascular compromise, and autonomic dysfunction converge to accelerate neurodegeneration. Importantly, this is not an isolated combination but a demonstration of how multiple organ systems interlock within the broader ecosystem framework, reinforcing the principle that resilience or collapse emerges from systemic integration rather than single-axis effects. Beyond microbial and environmental modulators, inter-individual pharmacogenetic variation also shapes susceptibility and treatment response, linking inherited differences in drug metabolism, transport, and immune signaling to axis-specific resilience or collapse.

### 6.3. Integration: Multi-Axis, Asynchronous Failures → Systemic Collapse

**Figure 4:**
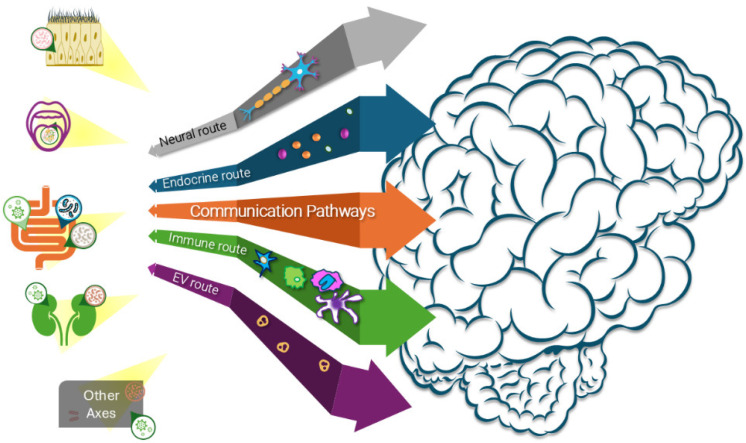
Multi-Route Communication in Organ–brain Axes. Peripheral organs communicate with the brain through multiple pathways: neural routes (vagus nerve, cranial nerves), endocrine signaling (hormones, metabolites), immune routes (cytokines, infiltrating cells), and extracellular vesicle (EV) trafficking carrying pathological cargo, including misfolded proteins and microbial components. Bidirectional communication allows brain pathology to propagate back to peripheral organs, establishing the feedback loops that characterize ecosystem failure. The convergence of multiple routes explains how peripheral dysfunction rapidly becomes systemic neurodegeneration.

The axes described are not isolated highways but interconnected circuits. The integration of these multiple communication pathways is depicted in Figure 4. As shown, peripheral organs do not communicate with the brain through a single route, but rather via convergent pathways that include neural conduits (vagus and cranial nerves), endocrine signaling (hormones and metabolites), immune routes (cytokines and infiltrating cells), and EVs trafficking, which carries both protective and pathological cargo. This multi-route architecture explains the redundancy that initially maintains homeostasis, and the vulnerability that enables rapid system-wide collapse once multiple axes begin to fail simultaneously. Collapse begins asynchronously: gut dysbiosis in one patient, renal stress in another (Table 2). However, over time, EVs, immune signaling, and descending neural tracts synchronize failures across systems [168]. This explains the heterogeneity of early clinical presentation and the convergence toward systemic multi-organ involvement. The emergence of tunneling nanotubes and glymphatic dysfunction as additional transmission routes adds further complexity to this network-level collapse, highlighting how multiple redundant pathways ensure pathological spread once ecosystem failure is initiated.

## 7. Persistence and Inheritance: Molecular Scars, Epiglial Scars, and Epigamilial Susceptibility

Whereas Section 5 outlined the acute synergistic pressures driving collapse, Section 7 examines how these insults leave durable scars that perpetuate dysfunction even after the initial triggers recede.

One of the most underappreciated aspects of neurodegeneration is its irreversible trajectory. Even when pathogens are cleared, inflammation subsides, or environmental toxins are reduced, patients rarely experience true regression. Instead, the disease proceeds inexorably, reflecting the capacity of biological systems to “remember” past insults in molecular and cellular form. This phenomenon is here conceptualized as molecular scars, proposed as long-lasting imprints that record prior exposures and bias tissues toward ongoing dysfunction. Within this broad framework, two more specific categories are proposed: epiglial scars, defined as a novel construct to describe the maladaptive reprogramming of glial cells within an individual’s lifetime, and epigamilial susceptibility, which is introduced as a concept to capture the intergenerational inheritance of pathogen- and environment-driven epigenetic marks. Together, these dimensions of memory explain why neurodegeneration persists across decades and why risk patterns cluster across families without clear genetic mutations.

### 7.1. Molecular Scars: Recording the History of Insults

The term molecular scars refers to the persistent biological memory of past pathogenic, toxic, or inflammatory events. They arise when acute stress leaves behind stable modifications that alter how cells and tissues respond to future challenges. While most synucleinopathies are sporadic, epidemiological studies suggest that environmental factors and epigenetic mechanisms influence disease risk. DNA methylation changes have been associated with MSA, and histone deacetylase SIRT1 plays a protective role against α-syn-induced neuronal death. Non-coding RNA families, including microRNAs (miRNAs), long noncoding RNAs (lncRNAs), and circular RNAs (circRNAs), regulate gene expression post-transcriptionally. Circulating exosomal miRNAs appear to be more dominant modulators of gene expression than free miRNAs, though their roles in MSA pathogenesis remain unclear [169]. Mechanistically, these scars often manifest as:

- Epigenetic reprogramming (DNA methylation, histone acetylation/methylation, long noncoding RNAs) that fix immune and glial cells into hyper-reactive states.
- Proteostatic templates, where misfolded intermediates seeded during earlier stress persist and act as nuclei for prion-like propagation.
- Mitochondrial and metabolic remodeling, where altered redox states and bio-energetics persist beyond the initiating insult, continually bias cells toward oxidative stress and energy deficits.

Importantly, molecular scars create a lagging shadow of pathology: even if the precipitating infection or toxin disappears, the cellular system does not revert to baseline. Instead, it carries forward the consequences of earlier exposures, lowering the threshold for protein misfolding and accelerating the transition into chronic neurodegeneration.

### 7.2. Epiglial Scars: The Persistence of Maladaptive Glial States

Within the CNS, molecular scars are particularly evident in glial populations: astrocytes, microglia, and oligodendrocytes that orchestrate homeostasis and response to injury [168]. These long-lasting maladaptive imprints are here defined as epiglial scars.

Glia are exquisitely sensitive to pathogens and inflammatory cues. Recurrent viral reactivations such as HSV-1 or EBV produce stable alterations in microglial chromatin landscapes, locking them into trained-immunity-like states [170]. Chronic oral and gut dysbiosis expose the CNS to waves of microbial amyloids and EVs, which prime microglia toward pro-inflammatory responses long after the initiating infection resolves [171]. Astrocytes exposed to persistent cytokine signals remodel their secretory and metabolic programs, diminishing their neuroprotective functions. Oligodendrocytes subjected to chronic interferon-γ signaling reduce myelin production and release exosomes enriched with pro-inflammatory or even misfolded proteins [172].

These scars do not merely reflect passive damage; they may constitute an active memory system of the CNS immune landscape. Even in the absence of ongoing infection, epiglial scars could sustain a milieu of oxidative stress, impaired proteostasis, and heightened sensitivity to secondary insults. This may explain why patients often continue to decline despite apparent clearance of pathogens or toxins: the glial network may have been reprogrammed into a state that favors misfolding, aggregation, and spread of α-syn, tau, or TDP-43.

### 7.3. Epigamilial Susceptibility: Intergenerational Inheritance of Scars

Persistence may not be confined to the timescale of an individual life. Increasing evidence suggests that pathogen- and environment-induced epigenetic modifications could potentially be transmitted across generations, a phenomenon proposed here as epigamilial susceptibility [173].

This phenomenon arises through several convergent mechanisms. First, germline cells can acquire epigenetic modifications in response to chronic infections, nutritional deprivation, or toxic exposures [174,175]. DNA methylation changes and minor RNA signatures in sperm have been linked to ancestral stress, metabolic syndrome, and infectious disease. Second, families typically share microbial and viral reservoirs: periodontal pathogens, dysbiotic gut microbiota, or latent herpes viruses, which continuously reinforce these marks across generations [176,177]. Finally, shared exposure to environmental stressors, such as heavy metals, pesticides, or air pollutants, compounds the effect, embedding disease susceptibility in biology and the environment [178,179]. The potential result is that familial clustering of neurodegenerative disease may reflect not only inherited genetic mutations but also epigamilial inheritance of scars. For example, a family with repeated exposure to oral HSV or to arsenic-contaminated water may transmit across generations both the pathogens and the epigenetic modifications they induce, biasing descendants toward maladaptive immune and proteostatic responses. This concept aligns with accumulating evidence for transgenerational epigenetic inheritance (TEI) in mammals, where environmental exposures in one generation influence disease risk in subsequent generations through heritable epigenetic modifications [174,180]. While TEI mechanisms in mammals remain an active area of investigation with ongoing debate about criteria and consistency, multiple studies have documented transgenerational effects of toxicants, nutritional stress, and pathogen exposure across animal models and human populations [175]. Recent analyses emphasize the need for rigorous criteria to distinguish intergenerational from truly transgenerational effects, which the epigamilial framework aims to address by incorporating both inherited epigenetic marks and continued family-level exposures. From this perspective, conditions traditionally labeled as “genetic” may instead represent complex hybrids of DNA sequence, epigenetic scars, and familial ecological continuity.

### 7.4. Memory Across Scales: Embedding Irreversibility

Distinguishing molecular scars as a general category and its two novel subtypes-epiglial scars (intralifetime) and epigamilial susceptibility (intergenerational)-captures the layered persistence of neurodegenerative risk. Epiglial scars explain why misfolding and inflammation remain active within an individual long after the trigger is removed. Epigamilial susceptibility explains why risk recurs across families without clear genetic causation. Together, they illustrate how neurodegeneration embodies biological memory across scales, ensuring progression and heritability.

From this perspective, the irreversibility of neurodegeneration is the predictable outcome of ecosystem memory: the body “remembers” its microbial, toxic, and inflammatory history, and those memories, scarred into glia, mitochondria, and germlines, dictate future vulnerability. This framework reinforces the argument that prevention and treatment must move beyond symptomatic management to family- and ecosystem-level interventions: targeting pathogens, rebalancing microbiomes, reversing epigenetic marks, and restoring resilience before scars are permanently etched into biology.

### 7.5. Can Molecular Scars Be Reversed?

Molecular scars differ from genetic mutations: DNA sequence is fixed, but epigenetic, proteostatic, mitochondrial, and microbial changes retain plasticity, inviting the question-how far can they be reversed?

Epigenetic scars are among the most plastic. In oncology, inhibitors of DNA methyltransferases and histone deacetylases have demonstrated the ability to reset malignant epigenetic programs, providing a proof of principle that maladaptive cellular memories can be rewritten [181]. Applied to neurodegeneration, such approaches may restore transcriptional balance in pathways governing proteostasis and mitochondrial function, reduce inflammatory priming of glia, and enhance synaptic resilience. More selective agents–bromodomain inhibitors, histone methyltransferase modulators, or CRISPR-based epigenetic editors-offer the possibility of precisely targeting maladaptive marks without global reprogramming [182,183]. Epigenetic remodeling is not confined to pharmacology; environmental inputs exert equally profound effects. Exercise increases histone acetylation at neurotrophic genes, dietary polyphenols reshape methylation landscapes, and intermittent fasting resets circadian chromatin dynamics [184,185,186]. Together, these findings suggest that epigenetic scars, though durable, remain reprogrammable through pharmacological and lifestyle interventions.

Proteostatic scars are more challenging but not irredeemable. Misfolded proteins such as α-syn, tau, and TDP-43 persist as seeds, acting as durable templates for further aggregation [187]. Their dismantling requires restoration of folding capacity and engagement of clearance pathways. Molecular chaperones can refold or disaggregate toxic intermediates, while autophagy enhancers stimulate bulk clearance of aggregates. Immunotherapies directed against extracellular seeds have shown promise in neutralizing propagation loops, and proteasome modulators may prevent ongoing accumulation. More experimental approaches, including disaggregase peptides, molecular tweezers, or engineered proteases, are designed not merely to halt seeding but to actively dismantle entrenched templates. These data suggest that even proteostatic scars, long considered the hallmark of irreversibility, may be therapeutically targeted under the right systemic conditions.

Mitochondrial and metabolic scars represent another durable form of memory, as chronic oxidative stress leaves behind oxidized mitochondrial DNA (mtDNA), damaged cardiolipin, and maladaptive transcriptional programs. Yet mitochondria also retain extraordinary plasticity. Mitochondria-targeted antioxidants, such as MitoQ and SS-31, restore the redox balance, while NAD+ precursors rejuvenate sirtuin-mediated signaling [188,189]. PGC-1α activation promotes mitochondrial biogenesis, and mitophagy inducers selectively cull damaged organelles. These pharmacological approaches are reinforced by lifestyle interventions, including exercise, fasting, and caloric restriction mimetics, which all drive robust mitochondrial renewal, even in aged tissues. Thus, mitochondrial scars are not permanent inscriptions but dynamic imprints that can be reshaped if systemic energy balance is restored. This principle is increasingly validated in clinical contexts, where targeted mitochondrial restoration strategies show measurable functional recovery, as discussed in later sections.

Microbiome and biofilm scars pose yet another layer of persistence. Dysbiosis transforms cooperative microbial communities into opportunistic consortia that perpetuate inflammation and amyloidogenic exposure. Reversal requires not only the elimination of pathogens but also the restoration of ecological collaboration. Probiotic and prebiotic approaches can reintroduce missing taxa, fecal microbiota transplantation has demonstrated resilience restoration in Parkinson’s models, and phage therapy offers precision targeting of pathogenic strains without collateral damage. Antifungal strategies, particularly those targeting *Candida* overgrowth, can be viewed as a means to rebalance microbial hierarchies, thereby reducing competitive displacement and restoring symbiotic functions. These observations reinforce that microbial scars are not static lesions but ecological states that can be rebalanced toward health when systemic pressures are corrected.

Epigamilial scars, transmitted across generations, pose the most significant challenge. Yet even these are not immutable. Germline epigenetic marks can be attenuated through preconception interventions such as nutritional optimization, toxin avoidance, and pathogen control in prospective parents. Family-wide microbiome interventions, including eradication of persistent viral reservoirs or dysbiotic gut ecologies, may prevent reinforcement of ancestral scars in offspring. Emerging tools of epigenetic editing, such as CRISPR/dCas9 fused to demethylases or acetylases, may one day allow direct reversal of maladaptive germline imprints. Such approaches extend the horizon of neurodegeneration prevention beyond individuals to entire lineages, reframing genetic counseling as an ecological and microbial counseling.

These examples suggest that reversibility exists along a spectrum rather than as a binary state. Early-stage scars, including subtle epigenetic marks, minor microbiome imbalances, or low-grade mitochondrial stress, are highly reversible with lifestyle and pharmacological modulation. Intermediate scars, such as entrenched proteostatic templates, trained immunity, or metabolic remodeling, demand multimodal strategies to overcome. Late-stage scars, by contrast, require coordinated ecosystem restoration to reopen closed regenerative windows.

A critical distinction emerges between Parkinsonism driven by active systemic stressors versus chronic protein aggregation. Cases of severe infection-associated Parkinsonism, including HIV encephalopathy, influenza, and chemotherapy-induced syndromes, have demonstrated reversal following pathogen clearance or removal of the triggering agent [190,191,192,193,194,195,196,197]. These cases establish that even advanced basal ganglia dysfunction can be reversed when the underlying systemic drivers are addressed. Importantly, this reversibility extends beyond infection-linked cases: interventions targeting ecosystem balance show benefit across the entire spectrum of synucleinopathies, whether initiated by genetic mutations (SNCA, LRRK2, GBA), environmental toxins, or pathogens [198,199,200,201]. All converge on shared axes of ecosystem dysfunction-immune, metabolic, and microbial-that dictate protein behavior and disease course.

Converging evidence from independent pathways reveals a more profound insight: ecosystem interventions modify disease trajectory even when α-syn overexpression is the primary driver. Preclinical studies demonstrate that microbial signals modulate α-syn-dependent pathology independent of protein burden. In α-syn-overexpressing mice (Thy1-α-Syn model), antibiotic treatment improved fine and gross motor function, and gut motility defects, demonstrating therapeutic benefit despite persistent protein overexpression [198]. Germ-free conditions reduced motor deficits, while the reintroduction of short-chain fatty acids reinstated pathology, implicating specific microbial metabolites in disease propagation. Thus, ecosystem modulation can alleviate symptoms without necessarily reducing protein load.

Neuroinflammation constitutes a second mechanistic pathway. NLRP3 inflammasome inhibition with MCC950 prevented α-syn pathology and dopaminergic neurodegeneration in progressive PD mouse models [199]. Anti-inflammatory treatment preserved neurons and altered aggregation patterns, indicating that inflammation shapes misfolding dynamics rather than merely responding to them. NLRP3-deficient mice exhibited reduced motor dysfunction and neurodegeneration in toxin-based models, accompanied by near-complete abolition of microglial recruitment [202]. These findings suggest that protein aggregation, seeding competence, and neurotoxicity are all modulated by the inflammatory milieu.

Mitochondrial restoration represents a third mechanistic pathway with both preclinical validation and human clinical evidence. Nicotinamide riboside (NR), a precursor of NAD+, ameliorated mitochondrial dysfunction in patient-derived neurons from individuals with GBA-associated Parkinson’s disease and improved motor function in *Drosophila* PD models [203]. Critically, the NADPARK Phase 1 clinical trial (2022) demonstrated that oral NR supplementation increased cerebral NAD+ levels, altered brain metabolism, and was associated with mild clinical improvement, alongside transcriptional upregulation of mitochondrial, lysosomal, and proteasomal function [204].

These findings converge on a paradigm-shifting insight: α-syn pathology is not an autonomous process but an ecosystem-dependent phenomenon. Its severity and trajectory are sculpted by systemic inflammation, metabolic state, and microbial ecology. Thus, therapeutic benefit can be achieved by restoring ecosystem equilibrium: mitigating pathogen burden, resolving inflammation, and re-energizing mitochondrial networks rather than by directly dismantling protein aggregates. Because misfolded proteins remain responsive to their systemic milieu, ecosystem restoration emerges as a unifying therapeutic axis across synucleinopathies, irrespective of whether the initiating insult is genetic, toxic, or infectious.

Perhaps most fundamentally, emerging evidence challenges the assumption that neuronal death represents an absolute boundary for recovery. Adult neurogenesis of dopaminergic neurons in the substantia nigra has been documented in rodents, with stem cells capable of generating new dopaminergic neurons throughout the lifespan [204,205,206]. While the rate of physiological neurogenesis is slow, it increases following injury. Theoretical calculations suggest the entire dopaminergic population could be replaced over a mouse’s lifespan. Neurogenesis is enhanced by the same ecosystem interventions that modify protein pathology: exercise increases BDNF expression, which promotes neurogenesis and dopaminergic neuron survival [207]. Meta-analysis of human PD studies confirms that exercise significantly increases BDNF blood levels, paralleled by improvements in motor symptoms [208,209,210,211].

This neurogenic capacity reframes the concept of irreversibility. The therapeutic window may remain open as long as progenitor cells retain the capacity to generate new neurons, with the critical question being not whether neurons are lost but whether the ecosystem environment permits their regeneration. Inflammation, oxidative stress, and metabolic dysfunction-the very stressors characterizing ecosystem collapse-suppress neurogenesis and create a hostile environment for newly generated neurons. Conversely, ecosystem restoration through exercise, anti-inflammatory interventions, and metabolic optimization may alleviate these constraints, allowing endogenous regenerative processes to gradually restore neuronal populations. Under this framework, irreversibility does not reflect an intrinsic limitation but an ongoing ecosystem failure that suppresses regeneration.

Recovery potential exists whenever the ecosystem can be shifted from a neurodegenerative to a neurogenic state. The neurogenic framework has direct implications for regenerative medicine strategies (Section 10.3). When endogenous neurogenesis proves insufficient due to extensive neuronal loss or overwhelming ecosystem dysfunction, exogenous cellular therapies become necessary. However, the same ecosystem stressors that suppress endogenous neurogenesis, trigger inflammation, cause metabolic failure, and lack neurotrophic support will also compromise the survival and integration of transplanted or reprogrammed cells. Thus, ecosystem restoration serves as a standalone therapeutic strategy and as an enabling platform for regenerative medicine, creating the permissive microenvironment required for cellular therapies to succeed in late-stage disease.

These examples reveal that irreversibility is not absolute but rather reflects the rarity of aligning sufficient interventions to overwhelm scar rigidity. In the ecosystem failure paradigm, the earlier such resets are attempted, the easier it is to remodel scars. The later they are tried, the more multimodal the strategy must be. Recovery, even in late disease, underscores the latent plasticity of the human system once pathogen burden is lifted, metabolism is rebalanced, and glial scars are reprogrammed. Molecular scars, therefore, should be viewed not as determinants of inevitability, but as precise therapeutic targets, highlighting the need for systemic, multi-axis interventions that reboot a failing ecosystem rather than repair isolated nodes.

#### Potential Cascade Dynamics in Scar Reversal

Although molecular scars often appear entrenched, their reversal could potentially follow cascade-like dynamics, where targeted interventions in one domain might propagate improvements across multiple interconnected systems (Table 3). Rather than demanding simultaneous repair of all scar types, strategic leverage points may unlock positive feedback loops that gradually restore resilience. 

The cascade dynamics described in Table 3 represent a theoretical framework synthesized from studies of individual pathways. The proposed positive feedback loops have not been systematically validated in human neurodegeneration, and the temporal sequences and threshold effects remain to be established via controlled studies.

One example is the epigenetic cascade: reducing pathogen burden or metabolic stress can reactivate deacetylases such as SIRT1, which in turn coordinates autophagy, dampens inflammatory gene expression, and enhances mitochondrial biogenesis [212,213,214]. These changes mutually reinforce, creating a self-sustaining transcriptional reset. Similarly, a microbiome-immune cascade can unfold when pathogenic biofilms are disrupted: recolonization by commensals produces short-chain fatty acids such as butyrate, which improve gut barrier function, reduce systemic cytokine load, and stabilize immune tolerance [215,216,217]. A parallel proteostatic cascade emerges once the aggregate burden drops below a critical threshold–chaperone networks regain capacity, accelerate aggregate clearance, and restore folding homeostasis in a self-amplifying manner. Finally, mitochondrial cascades can be triggered by reducing oxidative stress: new mitochondrial biogenesis improves energy status, which fuels ATP-dependent repair processes and further stabilizes proteostasis and immunity [218].

These cascades suggest that scar reversal functions more as a domino effect than an all-or-none process, where a timed intervention can restore systemic balance. However, this depends on first reducing microbial, viral, or toxin burden, as persistent stressors reinforce scars and block repair. Once ongoing insults cease, feedback loops in epigenetic, microbiome, proteostatic, and mitochondrial domains can gain traction. The existence of cascade dynamics provides a framework for understanding why pathogen control, fasting, exercise, or microbiome resets can sometimes yield disproportionately broad improvements across scar domains. However, claims of complete recovery in advanced neurodegeneration require cautious interpretation. While case reports of functional improvement exist, the evidence base remains limited and individual responses vary significantly. The complexity of coordinating multiple interventions and maintaining long-term adherence presents practical challenges that must be acknowledged alongside the theoretical potential for scar reversal.

Misfolded proteins may also persist in a silent state, as shown by α-syn deposits in the appendix of healthy individuals. Such reservoirs only become pathogenic when inflammatory environments reduce clearance capacity and enhance vesicle-mediated spread, unlocking their prion-like behavior. Importantly, prion-like dynamics can also apply to inflammation itself: chronic immune activation, whether maintained by latent viral reservoirs, trained immunity, or unresolved scars, propagates as a self-sustaining positive feedback loop. Latent viruses periodically probe the host environment by releasing toxins, proteins, or vesicles in short bursts; these pulses act as pathogenic “sparks” that sustain inflammatory and misfolding cascades even without full reactivation. In this sense, both protein misfolding and inflammopathy represent parallel forms of ecosystem memory, persisting beyond initial triggers and progressively amplifying systemic vulnerability.

## 8. Neurodegeneration as Ecosystem Failure: A New Paradigm

Traditional models of neurodegeneration have tended to localize pathology within the brain, framing disease as the product of discrete protein misfolding events, genetic mutations, or isolated toxic exposures. This reductionist view has generated important insights into protein biology but fails to capture the clinical reality: neurodegenerative diseases are systemic, heterogeneous, and persistently progressive, often defying narrow explanations. The cumulative evidence presented across this review suggests that these conditions are best understood not as single-organ failures, but as ecosystem failures–where microbial, immune, metabolic, and environmental networks collapse in synchrony, leaving behind persistent scars that perpetuate decline.

### 8.1. From Protein Misfolding to Network Collapse

At the molecular core of neurodegeneration lies the prion-like misfolding of proteins such as α-syn, tau, and TDP-43 [218]. These proteins exist not as static entities but as conformational ensembles that can be biased toward pathogenic strains by microbial amyloids, oxidative stress, or post-translational modifications. Once misfolded, they appear to propagate through EVs, synaptic connections, or descending tracts, potentially creating a self-sustaining cycle of pathology. Yet this cycle does not remain confined to the CNS. Pathological proteins cross the BBB primarily through EVs, which can traverse endothelial barriers and deliver misfolded cargo directly to CNS tissues, as detailed in Section 4.1. As described in Section 6, misfolded proteins are increasingly detected in peripheral tissues-gut, kidney, heart, skin, demonstrating that disease represents not just a brain pathology but a multi-organ syndrome.

The propagation of misfolded proteins across organ–brain axes may explain both the heterogeneity of symptoms and the convergence toward systemic decline. A patient whose disease potentially begins in the gut may first exhibit constipation and autonomic symptoms, while another whose pathology potentially originates in the kidney may present with toxin-sensitive parkinsonism. Ultimately, however, both trajectories converge on widespread misfolding, reflecting the collapse of resilience across interconnected organ ecosystems.

### 8.2. The Perfect Storm and the Failure of Buffering

After outlining how misfolded proteins propagate across organ–brain networks, attention turns to the systemic convergence of stressors, the “perfect storm,” that precipitates collapse. Neurodegeneration appears to rarely result from a single insult. Instead, it may reflect a perfect storm of microbial amyloids, viral reactivations, toxic exposures, and inflammatory cascades that collectively could overwhelm the host’s buffering capacity. In this framework, the “perfect storm” represents the systemic convergence of ecological stressors–pathogens, toxins, metabolic dysregulation, circadian disruption, and immune maladaptation, whose combined action drives proteostatic failure and accelerates prion-like propagation. It is not the presence of any single factor, but the synchrony and persistence of multiple insults that shift the system from a compensatory balance to irreversible collapse. Biofilms act as ignition zones, releasing amyloidogenic proteins and vesicles. Viral latency creates layered pulses of stress. Mast cells and microglia amplify inflammation through vesicle-based feedforward loops. Mitochondria, far from being passive victims, serve as hubs that translate microbial and immune signals into systemic redox stress. Environmental toxins-from heavy metals to pesticides-further erode proteostatic capacity, reshaping microbiomes in ways that reinforce amyloidogenic exposure.

The cumulative effect may represent a progressive exhaustion of resilience and is a systems-level threshold phenomenon rather than a simple linear accumulation. Individual stressors may remain below pathogenic thresholds, but their interaction creates emergent properties-oxidative cascades that amplify each other, immune priming that reduces clearance capacity, and metabolic shifts that favor misfolding. This may explain why identical exposures produce different outcomes depending on timing, genetic background, and concurrent stressors, supporting a personalized rather than uniform pathogenesis model. For decades, the system may appear to compensate, maintaining a precarious equilibrium. Eventually, however, a tipping point may be reached where buffering collapses, and the system transitions into self-propagating decline, potentially elucidating the long prodromal phases and the abrupt acceleration often observed in clinical practice.

### 8.3. Memory Layers: Scars That Embed Irreversibility

The persistence of neurodegeneration appears to be rooted in the formation of molecular scars. Epigenetic modifications, proteostatic templates, mitochondrial imprints, and microbial dysbioses constitute durable records of past exposures. Within this umbrella, epiglial scars are proposed to reflect maladaptive reprogramming of CNS glia and immune cells that sustain inflammation and misfolding across an individual’s lifetime, while epigamilial susceptibility is conceptualized as reflecting the intergenerational inheritance of pathogen- and environment-induced marks, potentially reinforced by shared exposures within families. These intertwined memories may explain the progression after triggers fade and familial clustering without clear genetic links. Importantly, scars are not wholly immutable. As discussed in Section 7.5, interventions ranging from epidrugs and chaperone inducers to microbiome transplantation and radical dietary resets can soften or even reverse scars, particularly when applied before the tipping point. Yet even in advanced disease, anecdotal reports of recovery suggest that systemic plasticity persists, awaiting conditions that allow scars to be remodeled. In this sense, irreversibility is not absolute but rather reflects the difficulty in synchronizing sufficient systemic resets.

### 8.4. Ecosystem Restoration: From Inevitability to Intervention

Framing neurodegeneration as ecosystem failure transforms the therapeutic outlook.

Recovery demands multimodal, systemic interventions targeting the disease ecology rather than isolated lesions, including:

- Pathogen control: manage viral latency, dismantle biofilms, rebalance microbial consortia.
- Epigenetic reprogramming: reverse maladaptive marks via selective drugs, editing tools, and lifestyle change.
- Proteostasis restoration: enhance chaperones, activate autophagy, dismantle aggregates.
- Mitochondrial renewal: revive bioenergetics through NAD^+^ boosters and mitophagy inducers.
- Ecological resets: shift diet, microbiome, and toxin load to resynchronize axes.
- Family-wide measures: address epigamilial risk and personalize therapy via pharmacogenetics [173].

In essence, restoration means rebooting the whole ecosystem–relieving pathogen load, rebuilding microbial cooperation, resetting energy metabolism, and retraining immune-glial memory.

### 8.5. A New Paradigm

Neurodegeneration, in this view, may be best understood as the systemic erosion of resilience across multiple organ–brain axes, potentially accelerated by pathogens, toxins, and environmental stressors, and possibly perpetuated by the scars they leave behind. It is not simply a disease of misfolded proteins, but rather a failure of ecosystems to reset, becoming locked into maladaptive trajectories. The corollary is that therapy must aim not at isolated lesions but at ecosystem restoration–a deliberate attempt to remodel scars, rebalance axes, and reestablish resilience.

This paradigm does not dismiss the importance of genetic mutations or protein biology, but it reframes them within a broader ecological context. Genes may confer susceptibility, and proteins may misfold, but whether disease emerges depends on the complex interplay of environmental factors, including microbes, toxins, immune responses, and scars, across various scales. Prevention and treatment, therefore, require a systems-level approach that integrates microbiology, immunology, metabolism, and epigenetics into a cohesive model of care.

## 9. Shared Mechanisms: Convergence Across Neurodegenerative Diseases–Ecosystem Failure Beyond Aggregates

Although the PSDs provide a compelling prototype for the ecosystem failure paradigm, the principles extend to all major neurodegenerative diseases. Each disorder reflects not only the spread of prion-like misfolded proteins, but also the loss of normal protein functions as these proteins become sequestered into aggregates. This functional collapse represents the true tipping point of disease: not the sheer burden of aggregates per se, but the emergence of “proteinopenias”- states of functional insufficiency that destabilize essential cellular processes across organs and brain. Table 4 summarizes the shared logic of protein functional insufficiency across neurodegenerative spectra, illustrating how distinct molecular failures converge on common ecosystem vulnerabilities. The consequences ripple outward via the organ-brain axes: impaired vesicle trafficking, microtubule destabilization, splicing dysfunction, and energy imbalance, each creating vulnerabilities that pathogens, toxins, and immune cascades exploit, amplifying systemic collapse. Thus, the unifying model is one of ecosystem failure driven by a perfect storm of insults, converging on protein functional insufficiency as the tipping point. In this light, the challenge for prevention and therapy is not merely to reduce aggregate load, but to preserve or restore the normal physiological functions of critical proteins, whether by stabilizing their conformations, supporting their interactions, or replacing their activity through regenerative strategies.

## 10. Toward Precision Ecosystem Medicine

Framing neurodegeneration as ecosystem failure transforms the therapeutic agenda. If disease emerges from a multi-axis collapse, perpetuated by the scars and functional insufficiency of critical proteins, then treatment cannot rely solely on single-target interventions. Instead, it must evolve into precision ecosystem medicine: a proposed systemic strategy that could integrate biomarkers, prevention, regenerative approaches, and computational stratification to restore resilience. Ecosystem restoration is not merely one therapeutic option among many, but rather the foundational requirement that enables all other interventions, including regenerative medicine, to succeed.

### 10.1. Biomarkers for Ecosystem Monitoring

Traditional biomarkers have focused on aggregates in CSF or imaging of discrete brain regions. Yet within an ecosystem paradigm, monitoring must capture dysfunction across multiple axes simultaneously. EVs represent particularly promising tools: they circulate between organs and the brain, carry signatures of protein misfolding, inflammatory state, and metabolic stress, and can be sampled in blood, saliva, or CSF, like a form of a “liquid biopsy”. Quantification of CNS-derived EVs in plasma and α-syn measurements in serum L1CAM-immunocaptured exosomes have emerged as novel biomarkers to distinguish between different synucleinopathies [219,220]. Skin biopsies have revealed peripheral α-syn and tau aggregates, accessible without invasive procedures. Gut microbiome profiling captures early shifts in amyloidogenic consortia and loss of metabolic symbionts. Plasma assays of cytokines, mitochondrial metabolites, and epigenetic marks further extend the biomarker space. Together, these tools enable a multi-axis portrait of disease activity, moving beyond single-protein measurements toward ecosystem surveillance. Multi-axis biomarker panels will likely require machine learning integration to identify patterns indicative of early ecosystem dysfunction before clinical symptoms emerge.

Table 5 addresses key mechanistic questions underlying the ecosystem failure paradigm and their implications for precision ecosystem medicine.

#### Clinical Applications: Illustrative Cases

To illustrate how precision ecosystem medicine might be applied in practice, the following hypothetical cases demonstrate how ecosystem profiling could guide individualized interventions. These examples are not intended as clinical evidence but as conceptual scenarios, highlighting how multi-axis assessment, biomarker integration, and ecological rebalancing strategies could alter disease trajectories across different patient profiles. Together, these scenarios illustrate how ecosystem profiling can transform from a theoretical construct into an actionable clinical strategy, guiding tailored interventions across different disease stages, multi-axis failures, and inherited susceptibilities.

**Case 1—Early Intervention**: A 58-year-old patient presents with mild cognitive fluctuations and constipation. Ecosystem profiling reveals elevated gut microbial EVs, reduced butyrate-producing taxa, and peripheral α-syn in skin biopsy. AI stratification identifies dominant gut–brain axis dysfunction. Intervention focuses on targeted probiotics, periodontal treatment, and intermittent fasting, delaying progression for more than three years.

**Case 2—Multi-axis Failure:** A 62-year-old with rapid parkinsonism exhibits kidney–brain axis stress (elevated uremic toxins, renal α-syn deposits), oral-brain involvement (*P. gingivalis*, *HSV-1* reactivation), and markers of trained immunity. Coordinated intervention includes antimicrobial therapy, heavy metal chelation, and antiviral prophylaxis, stabilizing the decline.

**Case 3—Familial Risk:** A 45-year-old asymptomatic individual with a family history shows epigamilial susceptibility markers and early epiglial scarring. Preventive ecosystem optimization prevents clinical onset over a ten-year follow-up.

### 10.2. Prevention and Interception in the Prodrome

The ecosystem model highlights the long prodromal phase of neurodegeneration, when early axis failures accumulate scars but before irreversible collapse. This window offers an opportunity for interception. Interventions aimed at microbial balance, such as periodontal hygiene, targeted probiotics, antifungal therapy, or phage-based modulation, may reduce amyloidogenic pressure. Antiviral strategies could suppress herpes virus or enterovirus reactivation that primes glial scars. Early clearance enhancers, such as low-dose autophagy activators or proteasome modulators, could potentially reduce proteostatic burden before seeds become entrenched. Nutritional strategies, such as polyphenol-rich diets, fasting-mimicking cycles, and ketogenic or Mediterranean regimens, can reinforce mitochondrial and epigenetic resilience. Collectively, these prodromal interventions aim to delay tipping points by reducing the cumulative insults that form molecular scars. Several can be applied immediately: comprehensive periodontal treatment for patients with early parkinsonism, clinically guided microbiome restoration, including appropriate antimicrobial or antifungal therapy when indicated by comprehensive stool analysis (recognizing that fungal overgrowth often signals underlying bacterial dysbiosis requiring broader ecological rebalancing), and structured lifestyle interventions combining intermittent fasting, polyphenol-rich nutrition, and circadian optimization. Long-term applications requiring further development include AI-driven ecosystem phenotyping, engineered exosome therapies, CSF pathogenic cargo clearance followed by healthy or therapeutic EV delivery, and epigenetic editing approaches [221]. This staged implementation allows immediate clinical benefit while building infrastructure for more sophisticated ecosystem interventions. These represent low-risk, high-reward strategies that align with the ecosystem framework, pending validation of more complex, multi-axis protocols. The timing of interventions may be critical, as some axes may be more amenable to restoration than others, depending on the stage of the disease.

### 10.3. Regenerative Strategies: Restoring Resilience

Even when scars are established, regenerative strategies may offer a pathway to restore function. Modulating exosomes and other EVs holds particular promise: healthy stem cell-derived vesicles could potentially deliver protective microRNAs, anti-aggregation proteins, and mitochondrial components to injured neural networks, possibly diluting or counterbalancing pathogenic vesicle traffic. Engineering stem cell niches to secrete tailored vesicle cargo could reinforce glial and neuronal repair. CSF nano-filtration systems (liquorpheresis), currently in experimental development, may provide a means to continuously remove pathogenic vesicles and misfolded seeds, thereby lowering systemic propagation pressure [222,223,224,225]. More broadly, regenerative medicine—including induced pluripotent stem cell-derived neurons, organoid-derived glia, and biomaterial scaffolds-offers avenues not only to replace lost cells but to re-establish network resilience, counteracting the functional insufficiencies of synucleinopenia, tauopenia, or TDP-43 depletion. The ecosystem failure paradigm reframes regenerative medicine not as an alternative to systemic intervention but as its natural extension for late-stage disease. The success of regenerative approaches depends critically on the biological environment into which they are introduced. Transplanting cells into an inflamed, metabolically compromised, toxin-laden ecosystem is analogous to planting seeds in contaminated soil: survival, integration, and function will be compromised regardless of cell quality.

Therefore, regenerative interventions must be embedded within an ecosystem restoration framework: first establishing a permissive microenvironment through anti-inflammatory strategies, metabolic optimization, microbiome rebalancing, and neurotrophic factor enhancement; then introducing cellular therapies into this prepared ‘soil’; and finally maintaining ecosystem health to support long-term graft survival and synaptic integration. This approach may explain the variable outcomes of past cellular therapy trials and suggests that future regenerative medicine protocols should incorporate systematic ecosystem assessment and optimization as prerequisites for cellular intervention. The goal is not to replace ecosystem medicine with cellular therapies but to leverage ecosystem restoration to maximize the therapeutic potential of regenerative approaches.

### 10.4. Precision Through Stratification and Artificial Intelligence Integration

The heterogeneity of neurodegeneration demands that interventions be stratified. Some patients may exhibit a dominant gut–brain axis collapse, others an immune–brain trajectory, or a primarily vascular-metabolic profile. Artificial intelligence (AI) can play a pivotal role in stratification, integrating multi-omics (genomics, epigenomics, proteomics, metabolomics), imaging, and longitudinal biomarker data to identify distinct ecosystem phenotypes. Machine learning models could potentially predict which axis is failing earliest, which scars are most entrenched, and which combination of interventions offers the highest probability of restoration. In this way, AI-enabled ecosystem profiling could replace the blunt diagnostic categories of today with dynamic maps of resilience and vulnerability, guiding tailored interventions across axes.

### 10.5. Roadmap to Ecosystem Restoration

Taken together, these approaches outline a roadmap for precision ecosystem medicine. Early detection through EVs, skin biopsies, and microbiome profiling enables axes surveillance, followed by prodromal interception to reduce microbial, viral and metabolic stress before scar formation. Regenerative therapies restore function after collapse, while AI integration could personalize strategies to the individual’s unique ecosystem profile. The unifying goal is not to eliminate single aggregates but to reboot the failing system by reducing pathogen and toxin burden, remodeling epigenetic and metabolic scars, restoring protein functions, and reestablishing organ–brain communication. Multi-center pilots and adaptive trials should validate ecosystem-guided approaches, using biomarkers for real-time feedback. Pharmacogenetics adds a critical layer-genetic variants in metabolism and immunity shape response-underscoring the need for integrated ecological and pharmacogenetic profiling. Family-wide interventions must address individual and epigamilial risk, reframing genetic counseling as ecological and microbial counseling.

### 10.6. Limitations and Future Directions

The ecosystem-failure paradigm, though integrative, still requires prospective validation. While individual components of the “perfect storm” model have experimental support, the proposed synergistic interactions remain to be tested systematically. Current evidence is strongest for binary interactions (e.g., specific virus-bacteria combinations), but the multi-pathogen convergence proposed here demands more complex experimental designs. Similarly, the cascade dynamics underlying scar reversal, while theoretically plausible based on individual pathway studies, need validation of the proposed positive feedback loops in integrated systems.

Novel constructs-synucleinopenia, epiglial scars, and epigamilial susceptibility-require validation to confirm clinical utility. While parallels exist in animal and human data, their specific application to neurodegeneration awaits longitudinal studies. Key biomarkers (e.g., organ-specific EVs, peripheral aggregates, cutaneous deposits) also lack cross-site standardization. Crucially, the paradigm assumes intact but overwhelmed defense systems-chaperones, autophagy, and lysosomal degradation, rather than their absence. Thus, neurodegeneration reflects a tipping point of defense saturation, not passive failure. These limitations underscore the gap between theory and application and define key directions for progress.

While the anatomical and functional connections among organ–brain axes, including their role as entry points and initiating sites of systemic propagation, are established, the next step is to experimentally validate causal interactions and intervention sequences within this ecosystem framework. The following areas represent critical near- and long-term directions for validating, operationalizing, and scaling the ecosystem-failure paradigm:

Biomarker standardization and validation: multicenter harmonization of EV assays, cutaneous and peripheral aggregate measures, and microbiome/metabolome panels; prospective cohorts with pre-registered analysis plans.

Causal testing of axes: interventional studies that deliberately reduce pathogenic/toxic burden first, then probe sequence-dependent effects on epigenetic, mitochondrial, proteostatic, and immune outcomes (testing the cascade prerequisite explicitly).

Latent Viral and Pathogenic Reservoirs: Anchors of Persistence and Potential Cascade Healing. Latent viral and microbial reservoirs may serve as ecological anchors that sustain inflammatory scars and propagate prion-like dynamics. Misfolded proteins can persist in a silent state–for example, α-syn deposits in the appendix of patients and healthy individuals. Such deposits are not inevitably pathogenic; instead, they become active when inflammatory environments reduce clearance capacity and enhance vesicle-mediated spread, unlocking their prion-like behavior. Similarly, inflammation can adopt prion-like properties: chronic immune activation, maintained by latent viral reservoirs, trained immunity, or unresolved scars, propagates as a self-sustaining positive feedback loop.

Latent viruses periodically probe the host environment by releasing toxins, proteins, or vesicles in short bursts. These “sparks” act as intermittent triggers that reinforce inflammatory and protein misfolding cascades even in the absence of full reactivation. In this sense, reservoirs function as systemic amplifiers of prion-like processes, maintaining vulnerability and preventing recovery. In principle, radical clearance of such reservoirs could accelerate healing cascades across multiple axes: if inflammation quiets, mitochondria can recover, proteostasis can reset, and the microbiome can stabilize. This represents the domino healing cascade in reverse. Yet whether such targeted interventions can tip the balance toward systemic resilience remains an open research question. Most viruses are challenging to eradicate, latency is often immunologically tolerated, and attempts at total clearance may cause unintended injury. Currently, there is no large-scale clinical evidence to support the notion that clearance of latency alone leads to cascade healing. However, indirect evidence exists. For example, CMV-seronegative transplant patients show improved immune recovery, suggesting that a reduced latent burden may create a more favorable environment for systemic repair.

Optimization of multimodal care: factorial or adaptive trials to determine order, dose, and duration of combined pathogen control, metabolic/circadian alignment, proteostasis support, and microbiome repair.

Adaptive/precision trial designs: Platform trials, aggregated frameworks, and synergy-mapping to handle heterogeneity and evolving biomarker readouts.

AI-enabled ecosystem profiling: integrative models that stratify patients, forecast tipping points, and personalize intervention sequences, linked to learning health-system pipelines.

Implementation science and policy: pathways for cross-disciplinary care, reimbursement models for multi-axis protocols, and governance frameworks that operationalize family-level screening and data integration within ethical and privacy standards.

Ethical and Policy Considerations. The transition to precision ecosystem medicine raises important ethical and policy questions that must be addressed in tandem with scientific advancements. The multi-generational nature of epigamilial susceptibility introduces complex considerations about family screening and intervention. If pathogen exposure or environmental toxins in one generation influence neurodegeneration risk in descendants, what are the ethical obligations for counseling, notification, and prevention?

Healthcare delivery must also adapt. Specialized silos will need to evolve into coordinated teams, prompting changes in training, reimbursement, and quality standards. Functional and environmental medicine may serve as transitional models, but require rigorous validation within an evidence-based framework to ensure efficacy and safety.

Privacy and data security are paramount as genomic, epigenomic, and microbiome profiles merge with environmental data. Such integration offers predictive power but also risks discrimination and inequity. Ensuring equitable access will be crucial in preventing the widening of health disparities. Finally, the shift toward family- and community-level care blurs traditional boundaries of individual medicine. Pharmacogenetic and pathogen data raise questions about consent, privacy, and autonomy, requiring public-health frameworks that balance personal rights with collective benefits.

## 11. From Ecosystem Failure to Precision Ecosystem Medicine

Neurodegeneration is no longer adequately explained by isolated proteinopathies or narrow genetic defects. Mounting evidence suggests that it arises from systemic ecosystem failure caused by interacting microbes, immune cascades, toxins, and metabolic stressors that converge into a “perfect storm” of insults. Within this storm, the prion-like propagation of misfolded proteins remains central, but irreversibility begins when their normal functions are lost. Thus, collapse stems not only from aggregate buildup but from erosion of vital protein functions.

Once resilience is lost, the system rarely recovers, because insults leave molecular scars, including epigenetic reprogramming, proteostatic templates, mitochondrial imprints, and microbial dysbioses, that persist across individuals and generations. At the individual level, epiglial scars reprogram glia and immune cells into maladaptive states that perpetuate inflammation and misfolding. At the familial level, epigamilial susceptibility potentially transmits pathogen- and environment-induced marks across generations, possibly explaining clustering of disease even in the absence of genetic mutations. Together, these scars shift neurodegeneration from an acute event into a memory-driven process locked into maladaptive loops.

Yet scars are not permanent. Reversibility exists along a continuum: epigenetic, mitochondrial, and microbial scars can be reshaped through autophagy, chaperones, or immunotherapy. Reports of late-stage recovery after systemic resets—dietary, microbial, or metabolic—challenge the dogma of irreversibility. Neurodegeneration thus represents a contest between scar accumulation and ecosystem repair.

The path forward lies in precision ecosystem medicine. EVs, microbiome, and plasma assays can map axis health; early interception of microbial or toxic stress may delay tipping points. Regenerative approaches, including exosome modulation, stem cell niches, and clearance of pathogenic cargo from CSF, followed by delivery of healthy or therapeutic EVs, offer avenues to restore resilience once collapse has begun. AI-driven, multi-omic integration may stratify patients into ecosystem phenotypes and tailor interventions that reboot failing systems.

In this view, the PSDs serve as a prototype, but the paradigm generalizes across all major neurodegenerative diseases. Each reflects ecosystem failure amplified by a perfect storm of insults, pivoting at functional insufficiency of key proteins, and perpetuated by molecular scars. Each also opens opportunities for prevention, prediction, and precision restoration. Neurodegeneration, long seen as an inexorable decline, can thus be reframed as a condition of ecosystem failure that is, at least in part, reversible through systemic restoration, a challenge to repair ecosystems rather than merely suppress symptoms.

Ultimately, the irreversibility of neurodegeneration is not absolute but reflects a self-sustaining positive feedback between scars, stressors, and systemic collapse. Degenerative loops appear to thrive in environments that continuously reinforce them, such as the presence of persistent pathogens, toxins, metabolic stress, or circadian disruption. Transitioning to a reversible state may require altering the environment so that it no longer favors degeneration but instead permits regenerative cascades. This take-home lesson reframes therapy: rather than targeting single nodes in isolation, interventions must reshape the ecological context to unlock the unlock the system’s latent capacity for resilience and recovery. Realizing this vision requires transforming medical practice from isolated specialty care to coordinated multi-disciplinary collaboration, where neurologists, gastroenterologists, infectious disease specialists, environmental medicine practitioners, immunologists, and systems biologists work as integrated teams to address the full spectrum of ecosystem dysfunction. While this review focuses on neurodegeneration, the ecosystem failure paradigm may also extend to other chronic, complex diseases that share similar features, including multi-organ involvement, microbiome–immune–metabolic interactions, prodromal phases, and heterogeneous phenotypes. Autoimmune disorders, metabolic diseases, and cardiovascular conditions all exhibit ecosystem-level dysfunction characterized by bidirectional organ-organ communication, inflammatory amplification, and molecular memory. The precision ecosystem medicine framework, which involves personalized multi-axis profiling guiding tailored interventions, represents a potentially transformative approach across chronic disease domains, warranting systematic investigation beyond neurodegeneration.

## Figures and Tables

**Table 2:** Organ–brain axes, entry points, and potential biomarkers.

Axis	Primary Stressors/Pathogens	Communication Routes	Misfolding Connections	Candidate Biomarkers	Memory Layer
Gut–Brain	Dysbiosis, curli-producing *E. coli*, *Candida albicans*, pesticides	Vagus nerve, microbial EVs, immune cytokines	α-Syn seeding in ENS; vagal spread; microbial amyloids cross-seeding host proteins	Stool microbial EVs, gut α-syn, vagal autonomic markers	Epiglial scars (trained microglia); Epigamilial (ancestral microbiome/lifestyle transmission)
Oral–Brain	*P. gingivalis*, *gingipains*, HSV/EBV latency	Cranial nerves (trigeminal), bloodstream, OMVs	Gingipain-mediated tau cleavage; α-syn misfolding; viral PTM effects	Salivary α-syn/tau; gingival EVs; oral microbiome shifts	Epiglial reprogramming from chronic inflammation; epigamilial from family-shared oral pathogens
Kidney–Brain	Uremic toxins, heavy metals (Pb, Hg, Cd, As), renal infections	Circulation, renal EVs, sympathetic renal nerves	α-Syn misfolding in renal tissue; proteostasis disruption via toxins	Plasma uremic toxins; renal EVs with misfolded α-syn	Epiglial scars from chronic toxin exposure; epigamilial via germline heavy-metal/epigenetic marks
Liver–Brain	Viral hepatitis, metabolic/lipid dysregulation, pollutants	Circulation, hepatocyte EVs, bile acid signaling	Tau hyperphosphorylation; α-syn aggregation from metabolic stress	Serum bile acids, hepatocyte EV cargo	Epiglial reprogramming of astrocytes; epigamilial via germline transmission of metabolic epigenetic marks
Heart–Brain	Autonomic dysfunction, cardiac infection, perfusion stress	Sympathetic nerves, systemic circulation	α-Syn inclusions in cardiac nerves; proteostasis stress via ischemia	MIBG scans, atrial biopsies, ECG markers	Epiglial scars (autonomic neuronal/glial reprogramming); minimal epigamilial component
Skin–Brain	Cutaneous microbes, fungal biofilms, toxins	Peripheral nerves, microbial EVs, bloodstream	α-Syn/tau aggregates in skin nerves and fibroblasts	Skin biopsy α-syn/tau; sweat gland markers	Epiglial scars (local nerve reprogramming); limited epigamilial unless familial exposures persist
Vagus–Brain	Enteric α-syn, microbial amyloids, toxins	Ascending/descending vagal tracts; immune signaling	Direct neuronal α-syn transport; propagation of misfolding up/down vagus	Vagal α-syn deposits; ENS α-syn	Epiglial scars (ENS + brainstem neurons); epigamilial only indirectly (shared exposures shaping vagal entry)
Immune–Organ–Brain (meta-axis)	Chronic infections, trained immunity, mast cell activation, systemic inflammation	Cytokines, infiltrating immune cells, immune EVs	Epiglial priming of microglia; systemic bias toward aggregation	Circulating cytokines; immune EV proteome	Central node-epiglial scars dominate (trained immunity, maladaptive glial states); epigamilial susceptibility integrates ancestral immune exposures

**Table 3:** Cascade dynamics in scar reversal.

Scar Type	Trigger of Reversal	Feedback/Amplification Loop	Supporting Evidence
Epigenetic scars	Reduction in pathogen burden; activation of SIRT1	Demethylation → improved autophagy → lower inflammation → restored mitochondrial homeostasis	Fasting/exercise improves autophagy, mitochondrial biomarkers
Microbiome scars	Biofilm disruption; recolonization with commensals	Butyrate/SCFAs → improved barrier → reduced cytokines → stable immunity	Probiotics/antibiotics normalize inflammatory markers in weeks
Proteostatic scars	Lowering the toxic protein burden below the threshold	Restored chaperones → clearance of aggregates → proteostasis recovery	Chaperone induction improves clearance in preclinical PD models
Mitochondrial scars	Reduced oxidative stress; biogenesis triggers	More ATP → supports proteostasis, autophagy, repair → systemic energy renewal	Exercise and NAD+ boosters show multi-system benefits

**Table 4:** Protein functional insufficiency (“proteinopenia”) as a unifying mechanism across neurodegenerative diseases. The table summarizes how distinct neurodegenerative disorders converge on a shared pathophysiological motif: sequestration of critical proteins into aggregates causes loss of their physiological functions, destabilizing systemic homeostasis. Each disease reflects a unique entry point within the broader ecosystem-failure paradigm, with downstream amplification across organ–brain axes.

Disease/Spectrum	Core Proteins	Physiological Functions	Pathogenic Shift (“Proteinopenia”)	Systemic Consequences/Axes Affected
PSDs (PD, MSA, PSP, LBD)	α-Syn	Synaptic vesicle cycling, dopamine regulation, neuronal plasticity	Synucleinopenia: loss of soluble α-syn due to sequestration in Lewy bodies	Network instability, dopaminergic depletion, gut–brain propagation
AD	Tau, Amyloid-β	Microtubule stabilization, axonal transport, synaptic maintenance	Tauopenia: loss of functional tau; Aβ-mediated stress amplifies seeding	Axonal transport defects, vascular and immune dysregulation, gut–brain link
ALS/FTD	TDP-43, SOD1, FUS	RNA processing, oxidative defense, DNA/RNA repair	TDP-43 depletion: nuclear loss from cytoplasmic aggregation; SOD1/FUS loss impairs redox homeostasis and RNA trafficking	Transcriptome instability, oxidative stress, skeletal and visceral organ involvement

**Table 5:** Key mechanistic questions in ecosystem failure.

Question	Primary Mechanisms	Supporting Evidence	Clinical Implications
How do peripheral aggregates cross the BBB?	EVs (exosomes, microvesicles) traverse endothelial barriers; misfolded proteins packaged in vesicles bypass BBB selectivity; inflammatory conditions increase BBB permeability	EVs from PD patient plasma induce α-syn aggregation in mice; CNS-derived EVs detectable in peripheral blood; microglia-derived EVs carry pathological cargo	EV-based biomarkers for early detection; therapeutic targeting of vesicle trafficking; BBB modulation strategies
What determines brain-first vs. periphery-first pathology?	Individual susceptibility factors: genetic variants affecting barrier integrity; local pathogen burden (oral, gut, renal); environmental exposures; stochastic seeding events	Vagotomy reduces PD risk; genetic forms often brain-first; sporadic cases show varied entry points; family clustering without clear mutations	Personalized risk assessment based on axis vulnerability; targeted prevention strategies; early intervention at failing axes
How does strain selection occur?	Ecological conditions favor specific conformational variants: inflammatory cytokines promote β-sheet strains; oxidative stress selects metal-binding variants; microbial amyloids template specific misfolding patterns	MSA vs. PD strains show distinct seeding properties; prion strain diversity determines clinical phenotype; environmental factors correlate with disease subtypes	Strain-specific therapies; environmental modification to prevent aggressive strains; biomarker-guided treatment selection
Why do some interventions fail while others succeed?	Timing relative to tipping point; completeness of multi-axis targeting; individual scar burden and reversibility; intervention synchronization across domains	Early interventions more effective; single-target approaches often fail; case reports of multi-modal success; scar plasticity varies by stage	Multi-axis intervention protocols; biomarker-guided timing; personalized intervention intensity based on scar assessment

## Data Availability

No new data were created or analyzed in this study. Data sharing is not applicable to this article.

## Supplementary Materials

The following supporting information can be downloaded at: https://www.mdpi.com/article/10.3390/ijms262211207/s1, [13,15,226,227,228,229,230,231,232,233,234,235,236,237,238,239,240,241].
