# Correction: Velikic et al. Neurodegeneration as Ecosystem Failure: A New Paradigm for Prevention and Treatment. *Int. J. Mol. Sci.* 2025, *26*, 11207

**DOI:** 10.3390/ijms262412153

**Published:** 2025-12-18

**Authors:** Gordana Velikic, Gordana Supic, Dusica L. Maric, Miljan Puletic, Maja Ovcak Kos, Danilo Vojvodic, Dusan M. Maric

**Affiliations:** 1 Hajim School of Engineering, University of Rochester, Rochester, NY 14627, USA; ducamaric@gmail.com; 2 Department for Research and Development, Clinic Orto MD-P.A.R.K.S. Hospital, 21000 Novi Sad, Serbia; 3 Institute for Medical Research, Military Medical Academy, 11000 Belgrade, Serbia; gogasupic@gmail.com (G.S.); vojvodic.danilo@gmail.com (D.V.); 4 Medical Faculty of Military Medical Academy, University of Defense, 11000 Belgrade, Serbia; 5 Department of Anatomy, Faculty of Medicine, University of Novi Sad, 21000 Novi Sad, Serbia; 6 Faculty of Stomatology, Pancevo, University Business Academy, 26000 Pancevo, Serbia; miljenko.puletic@gmail.com; 7 Faculty of Law, University of Nova Gorica, 5000 Nova Gorica, Slovenia

In the original publication [1], there was a mistake in Figures 2 and 3 as published. During the final file-assembly stage, a draft placeholder version of Figures 2 and 3 was inadvertently uploaded instead of the finalized figures intended for publication. The published draft contains visual inconsistencies that do not fully correspond to the manuscript description and may lead to misinterpretation of the illustrated processes. The corrected Figure 2 and Figure 3 appear below.

Additionally, there was a mistake in the legend for Figure 2. The original caption did not include a description of the upper plot in Figure 2, which was added at the Academic Editor’s request. This may reduce clarity, given the refined layout of the corrected figure. The correct legend appears below. The authors state that the scientific conclusions are unaffected. This correction was approved by the Academic Editor. The original publication has also been updated.

**Figure 2:**
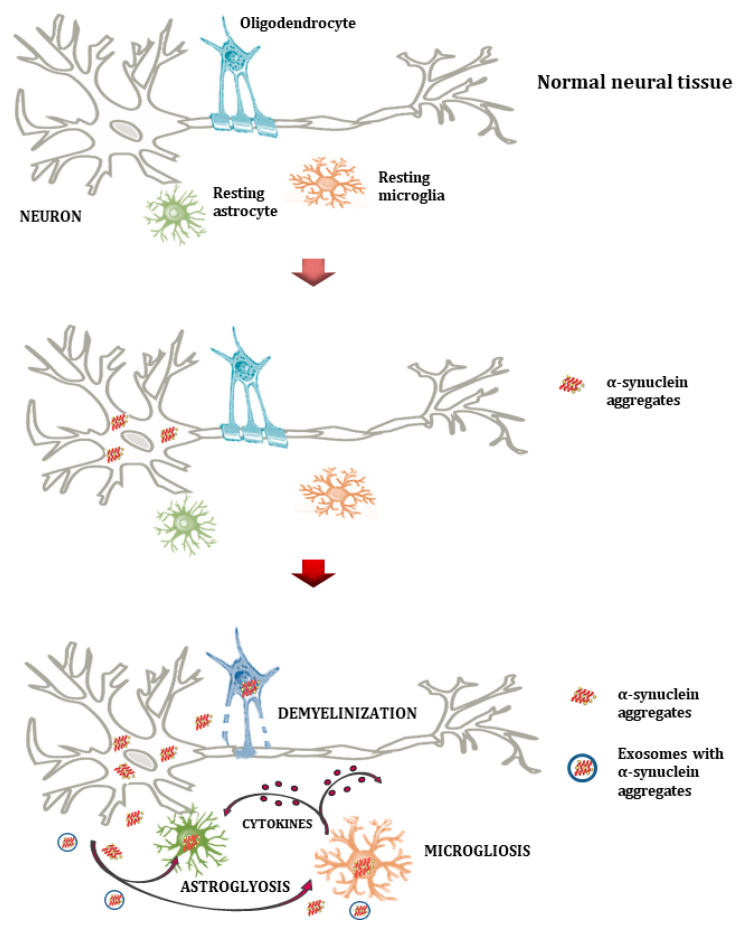
Normal neural tissue (**upper plot**) illustrates healthy interactions among neurons, oligodendrocytes, astrocytes, and microglia. Aggregates of α-syn form through the induced expression of the α-syn gene (SCNA) by various cells and/or the ineffective glial clearance mechanism (**middle plot**). Microglia and astrocytes phagocytize and accumulate misfolded α-syn. This results in oligodendrocyte degeneration, the loss of trophic support to neurons, and demyelination (**lower plot**). Extracellular α-syn aggregates cause astrocytosis and microgliosis, as well as the release of pro-inflammatory cytokines and reactive oxygen species, creating a neuroinflammatory milieu that further impairs neuronal integrity in PSDs. Microglia appear to have a dual role that involves the removal of α-syn and the production of cytokines.

**Figure 3:**
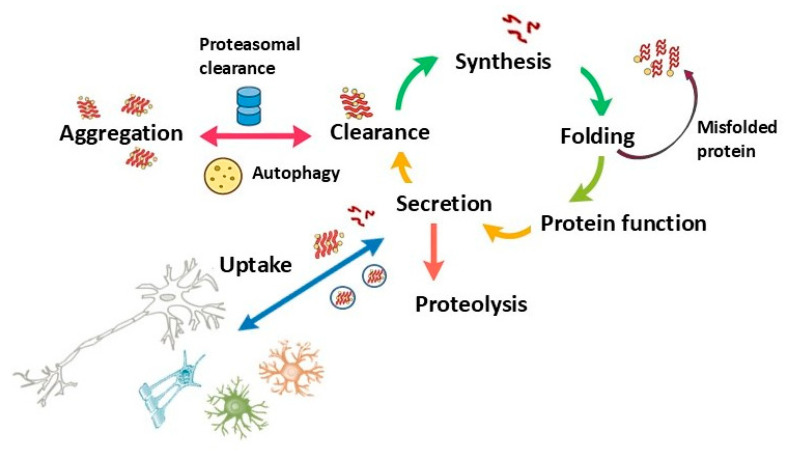
Protein Homeostasis Cycle: This schematic represents the cyclical processes involved in protein synthesis, folding, function, secretion, and clearance. Dysregulation at any point, particularly in folding, aggregation, or clearance, can lead to protein misfolding and aggregation, contributing to neurodegenerative processes. Uptake by surrounding cells highlights the potential for intercellular spread of misfolded proteins, a feature relevant in diseases such as MSA and PD, where protein aggregation and propagation exacerbate cellular dysfunction.
