# Supplementary material for: Neurodegeneration as Ecosystem Failure: A New Paradigm for Prevention and Treatment
Source: Int J Mol Sci. 2025 Nov 20;26(22):11207. doi: 10.3390/ijms262211207 (PMC12652886; doi:10.3390/ijms262211207)
Supplement: Supplementary file 1 [file ijms-26-11207-s001.zip › ijms-3890716-supplementary.pdf]

Table S1. Comprehensive comparison of MSA, PD, PSP, and LBD, integrating symptoms, treatments, and key distinctions in the pathophysiology [13,15,226–241]. The table combines clinical and mechanistic perspectives, outlining shared features, distinctive red flags that aid classification, and key pathophysiological differences. These dimensions together underscore how variations in protein aggregation, neuronal targets, and systemic involvement may correspond to distinct modes of ecosystem failure.

	Clinical Overview			Pathophysiological Overview		
**Disease**	**Shared Symptoms**	**Distinguishing Features**	**Other Clinical Features/Management**	**Protein Aggregation & Neuronal Targets**	**Pathophysiological Modifiers**	**Etiology**
**MSA**	Parkinsonism; autonomic dysfunction; ataxia	Early severe autonomic failure; poor response to PD meds; stridor	Rapid progression; cold, clammy skin; severe sleep apnea; speech therapy beneficial; bladder meds often required	α-syn in oligodendrocytes ⟶ glial cytoplasmic inclusions; widespread basal ganglia, cerebellum, brainstem	Strong neuroinflammation; severe autonomic failure	Mostly sporadic; some genetic risks; undefined environmental triggers
**PD**	Tremor, bradykinesia, rigidity; parkinsonism	Tremor at rest; good levodopa response	Hyposmia; micrographia; daytime sleepiness; DBS in selected cases; bladder/voice issues later	α-syn in neurons ⟶ Lewy bodies; dopaminergic loss in substantia nigra	Milder neuroinflammation; moderate autonomic involvement	Sporadic; LRRK2, SNCA mutations; pesticides/environmental links
**PSP**	Parkinsonism; poor coordination; impaired eye movements	Early balance problems; vertical gaze palsy	Personality change; dysphagia; frequent falls; speech slow/slurred; bladder less affected	Tau in neurons and glia ⟶ neurofibrillary tangles; basal ganglia & brainstem targets	Inflammation around tau deposits; less autonomic dysfunction	Largely sporadic; some genetic risk factors
**LBD**	Parkinsonism; cognitive fluctuations; hallucinations	Early cognitive decline; prominent hallucinations/delusions	Capgras syndrome; REM sleep disturbance; cholinesterase inhibitors helpful; bladder manageable with meds	-syn in cortical & subcortical neurons ⟶ Lewy bodies	Synaptic dysfunction; variable autonomic symptoms	Similar to PD; genetic predisposition; environmental links less defined
